# Metaproteogenomic analysis of saliva samples from Parkinson’s disease patients with cognitive impairment

**DOI:** 10.1038/s41522-023-00452-x

**Published:** 2023-11-18

**Authors:** Muzaffer Arıkan, Tuğçe Kahraman Demir, Zeynep Yıldız, Özkan Ufuk Nalbantoğlu, Nur Damla Korkmaz, Nesrin H. Yılmaz, Aysu Şen, Mutlu Özcan, Thilo Muth, Lütfü Hanoğlu, Süleyman Yıldırım

**Affiliations:** 1 Regenerative and Restorative Medicine Research Center (REMER), Research Institute for Health Sciences and Technologies (SABITA), Istanbul Medipol University, Istanbul, Türkiye; 2 Department of Medical Biology, International School of Medicine, Istanbul Medipol University, Istanbul, Türkiye; 3 Department of Electroneurophysiology, Vocational School, Biruni University, Istanbul, Türkiye; 4 Department of Psychology, Faculty of Humanities and Social Sciences, Fatih Sultan Mehmet Vakif University, Istanbul, Türkiye; 5 Department of Computer Engineering, Erciyes University, Kayseri, Türkiye; 6 Genome and Stem Cell Center (GenKok), Erciyes University, Kayseri, Türkiye; 7 Neuroscience Graduate Program, Istanbul Medipol University, Istanbul, Türkiye; 8 Department of Neurology, Istanbul Medipol University Training and Research Hospital, Istanbul, Türkiye; 9 Department of Neurology, Bakırkoy Research and Training Hospital for Psychiatric and Neurological Diseases, Istanbul, Türkiye; 10 Division of Dental Biomaterials, Center for Dental Medicine, University of Zurich, Clinic for Reconstructive Dentistry, Zurich, Switzerland; 11 Section eScience (S.3), Federal Institute for Materials Research and Testing (BAM), Berlin, Germany; 12 Department of Medical Microbiology, International School of Medicine, Istanbul Medipol University, Istanbul, Türkiye; 13 Present Address: Department of Medical Biology, School of Medicine, Bezmialem Vakif University, Istanbul, Türkiye

**Keywords:** Next-generation sequencing, Microbial communities, Clinical microbiology

## Abstract

Cognitive impairment (CI) is very common in patients with Parkinson’s Disease (PD) and progressively develops on a spectrum from mild cognitive impairment (PD-MCI) to full dementia (PDD). Identification of PD patients at risk of developing cognitive decline, therefore, is unmet need in the clinic to manage the disease. Previous studies reported that oral microbiota of PD patients was altered even at early stages and poor oral hygiene is associated with dementia. However, data from single modalities are often unable to explain complex chronic diseases in the brain and cannot reliably predict the risk of disease progression. Here, we performed integrative metaproteogenomic characterization of salivary microbiota and tested the hypothesis that biological molecules of saliva and saliva microbiota dynamically shift in association with the progression of cognitive decline and harbor discriminatory key signatures across the spectrum of CI in PD. We recruited a cohort of 115 participants in a multi-center study and employed multi-omics factor analysis (MOFA) to integrate amplicon sequencing and metaproteomic analysis to identify signature taxa and proteins in saliva. Our baseline analyses revealed contrasting interplay between the genus Neisseria and Lactobacillus and Ligilactobacillus genera across the spectrum of CI. The group specific signature profiles enabled us to identify bacterial genera and protein groups associated with CI stages in PD. Our study describes compositional dynamics of saliva across the spectrum of CI in PD and paves the way for developing non-invasive biomarker strategies to predict the risk of CI progression in PD.

## Introduction

Parkinson’s disease (PD) is a complex neurodegenerative disorder estimated to affect over 6 million people worldwide^1^. Due to the impact of the ageing population, a considerable increase in PD cases is expected in thefuture decades^2^. PD physiopathology is attributed to the alpha-synuclein (α-syn) aggregates accumulating in the neurons, which causes significant disruption of both motor and non-motor functions in the course of the disease^3^. One of its most common non-motor symptoms is cognitive impairment (CI) that progressively develops on a spectrum from mild cognitive impairment (MCI) to full-scale dementia (PDD)^4^. Despite variability among patients, there is a high risk of dementia in PD, with nearly half of patients reaching the dementia stage within 10 years after diagnosis and virtually all patients develop full dementia within 20 years after diagnosis^5^. These patients cannot live independent lives and require care and support from their families and nursing homes, leading to economic burden. Thus, the current unmet need in the clinic is whether the PD patients at risk of developing cognitive decline can be predicted in order to implement disease changing interventions.

Saliva is a complex biofluid and considered to be a rich source of potential biomarkers for chronic diseases as saliva components typically include host cells, microbiota and biological molecules^6^. The oral health of PD patients such as saliva secretion, the composition of saliva, and dysbiosis significantly aggravate in the course of the disease^7^. Indeed, α-Syn can be detected in different biological fluids, including cerebrospinal fluid (CSF) and saliva^8–10^. The alpha-synuclein pathology in the oral cavity of PD patients often leads to poor secretion of saliva and dysphagia^11,12^. Remarkably, a 6-year follow-up study of dysphagia in PD patients reported a significant association between CI and dysphagia^13^. Another common oral motor disorder of PD is drooling which has also been associated with CI in PD^14^. Together, these findings suggest a link between oral problems and CI in PD. Furthermore, recent studies reporting oral microbiota dysbiosis of PD patients linked dysphagia, drooling, and salivary pH with oral microbiota^15,16^. Therefore, we hypothesized that biological molecules of saliva and saliva microbiota dynamically shift in association with CI progression in PD and harbors discriminatory key signature changes for predicting CI stages in PD.

To test the hypothesis, we employed metaproteogenomics approach by integrating 16 S rRNA gene amplicon sequencing and metaproteomics data generated from saliva samples. We recruited 115 subjects to identify changes in saliva composition that can be used to differentiate PD patients at different CI stages. We determined that salivary microbiota differentiates CI stages and detected bacterial taxa associated with CI. We also identified functional level changes associated with CI in PD, and highlighted a short list of candidate signatures differentiating CI stages in PD.

## Results

### Characteristics of participants and analyses

**Fig. 1:**
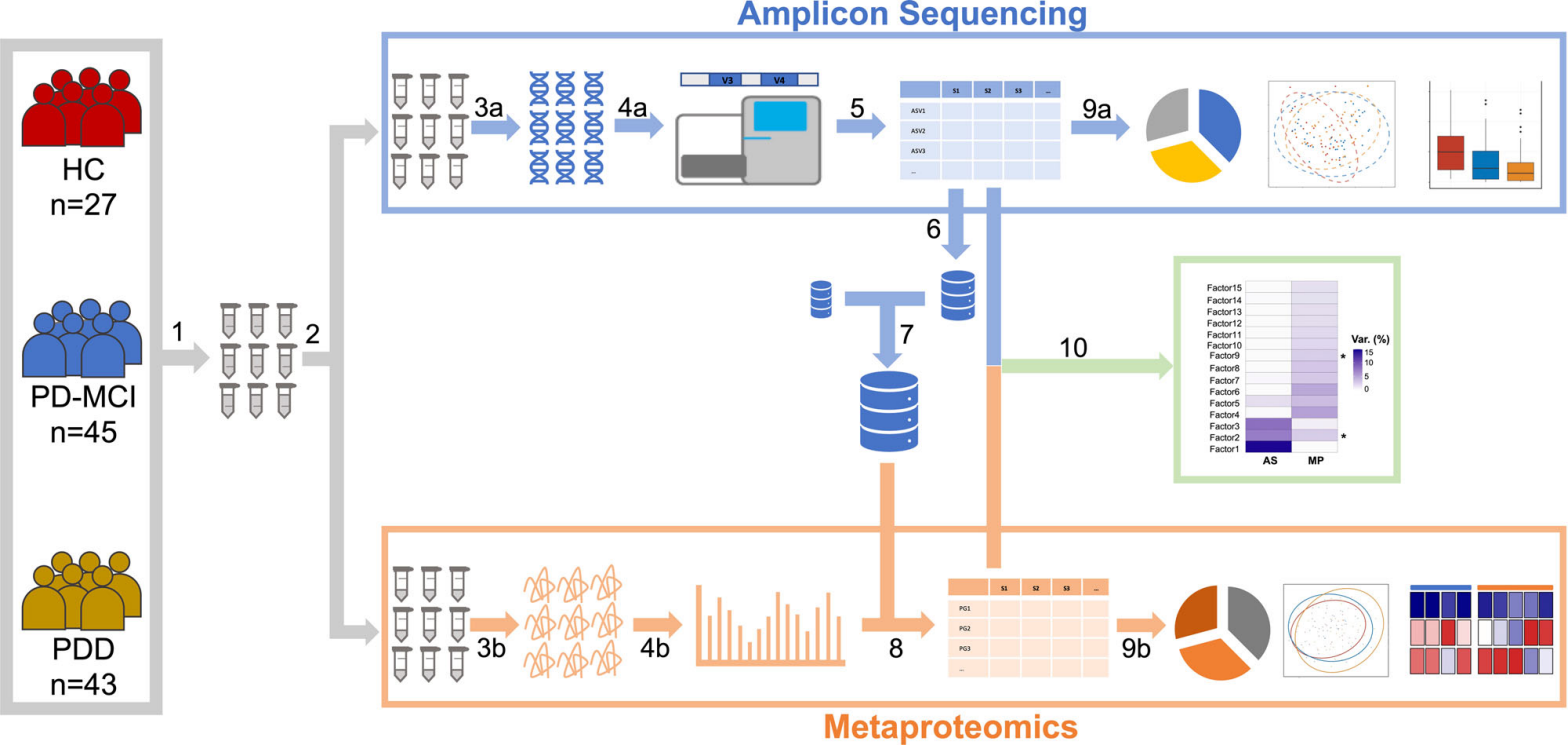
Experimental overview. (1) Saliva samples were collected from a total of 115 individuals (43 PDD, 45 PD-MCI) and 27 HC. (2) Samples were divided into two equal aliquots and used for DNA and protein extractions separately. (3a,b) DNA and protein isolations were performed. (4a,b) Amplicon libraries and tryptic peptides were prepared for NGS and LC-MS/MS, respectively. (5) ASV abundances were determined. (6) Predicted bacterial proteins of species-level genome bins that belonged to the 20 most abundant genera and bacterial taxa that showed differential abundance between study groups in amplicon sequencing were determined. (7) All human proteins from UniProt database were added to the predicted bacterial proteins which produced a final protein database that was used for protein identifications. (8) Protein group abundances were determined. (9a,b) Analyses of amplicon sequencing and metaproteomics data were performed. (10) Amplicon sequencing and metaproteomics datasets were integrated using MOFA.

A total of 115 individuals (43 PDD, 45 PD-MCI) and 27 HC were included in this study. Both 16 S rRNA gene amplicon sequencing based microbiome analysis and metaproteomics profiling were performed for all salivary samples collected from the participants (Fig. 1).

The demographic and clinical features of the study participants are summarized in Table 1. The mean age differed significantly between the study groups. We therefore adjusted all *P*-values for Age confounder, where appropriate. There was no significant difference in the proportion of female subjects between study groups. Years of education differed significantly between PD-MCI and HC, PDD and HC but not between PD-MCI and PDD participants which was also adjusted. There was expectedly a significant difference in mean MMSE scores pairwise between all study groups. The HC group had a mean MMSE score of 27.9, MCI group had a mean MMSE score of 23.6, and PDD group had a mean MMSE score of 18.7.

**Table 1:** Demographic and clinical features of the study cohort.

Characteristics	HC	PD-MCI	PDD
	(*n* = 27)	(*n* = 43)	(*n* = 45)
Age (years, mean ± SD)	59.6 ± 8.20	67.1 ± 8.48^1^	71.4 ± 7.10^1,2^
Sex (Female)	15 (55.6%)	17 (39.5%)	21 (46.7%)
Education (years, mean ± SD)	10.8 ± 5.1	7.4 ± 4.8^1^	4.6 ± 4.5^1^
MMSE (mean ± SD)	27.9 ± 1.9	23.6 ± 2.9^1^	18.7 ± 3.6^1,2^
CDR (mean ± SD)	0.0 ± 0.0	0.5 ± 0.0^1^	1.2 ± 0.5^1,2^
Hoehn and Yahr score (mean ± SD)	–	1.9 ± 0.8	2.6 ± 0.9^2^
UPDRS-part II (mean ± SD)	–	32.3 ± 14.9	50.0 ± 16.8^2^
PD Duration (months) (mean ± SD)	–	68.9 ± 44.5	105.2 ± 56.9^2^

*PD* Parkinson’s Disease, *SD* Standard Deviation, *MMSE* Mini-Mental State Examination, *HC* Healthy Control, *PD-MCI* Parkinson’s Disease with Mild Cognitive Impairment, *PDD* Parkinson’s Disease with Dementia.

^1^*p* < 0.05 for pairwise comparison with HC.

^2^*p* < 0.05 for pairwise comparison with PD-MCI.

Comparison of 16 S rRNA based and metaproteomics based microbial composition results

We performed both 16 S rRNA gene amplicon sequencing and metaproteomics for all 115 salivary samples. First, we compared ASV-based taxonomic composition with metaproteomics based taxonomic composition.

**Fig. 2:**
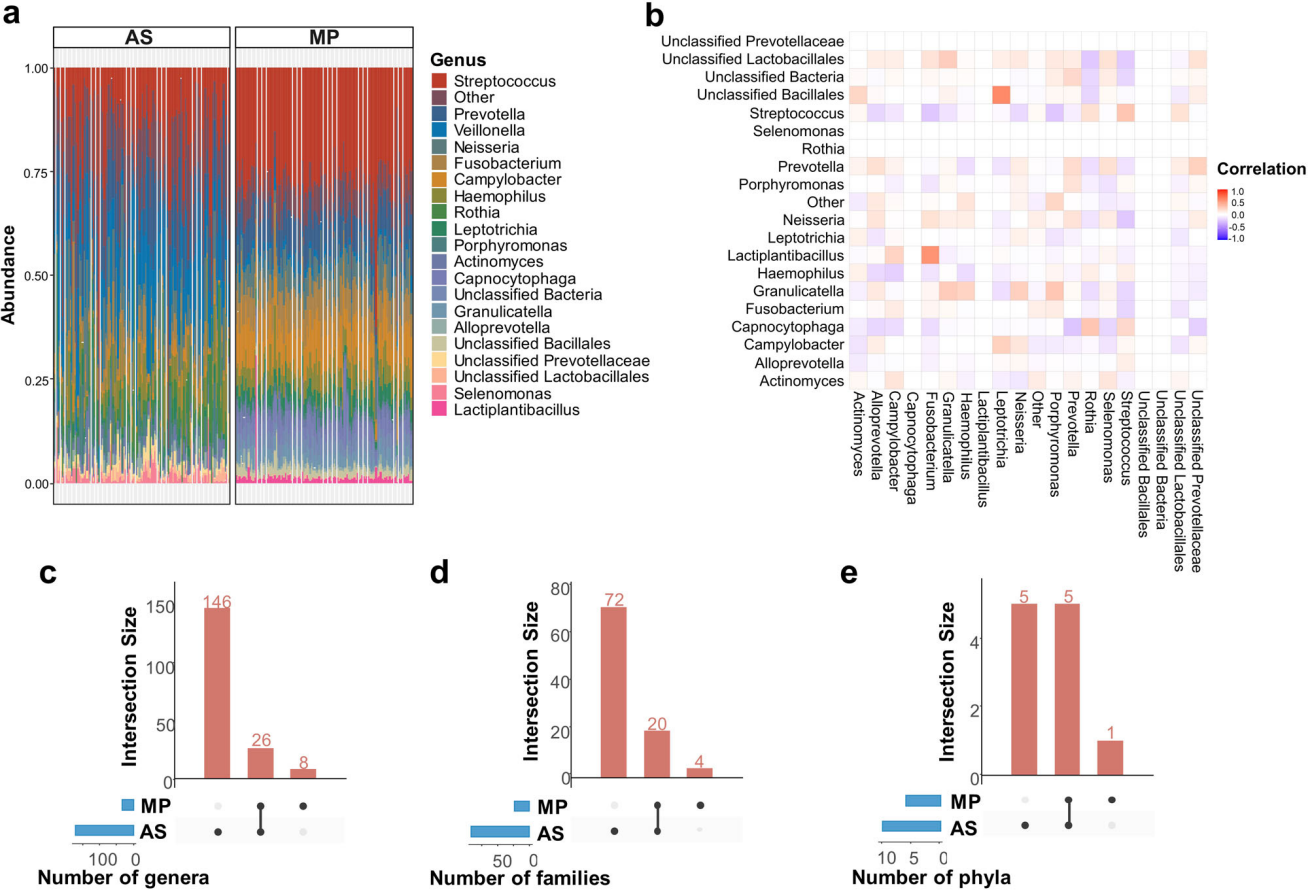
16 S rRNA gene amplicon sequencing (AS) and metaproteomics (MP) based overview of salivary microbiota composition across samples. **a** The 20 most common bacterial genera in salivary microbiota samples by 16 S rRNA gene amplicon sequencing and metaproteomics. Genera that were not among 20 most common taxa were grouped into “Other.” Each bar represents relative abundance distribution for a sample. The order of sample bars is the same for both methods. **b** Correlation between abundance of bacterial genera by 16 S rRNA gene amplicon sequencing versus metaproteomics. Verticle axis shows bacterial genera by 16 S rRNA gene amplicon sequencing while horizontal axis shows bacterial genera by metaproteomics. **c**–**e** Number of genera, families and phyla found across 16 S rRNA gene amplicon sequencing and metaproteomics. Vertical bars represent the number of taxa shared between two methods highlighted with connected dots in the lower panel. The horizontal bars in the lower panel indicate the total number of taxa detected by each method.

The 5 most abundant genera in salivary microbiota samples across sample groups were *Streptococcus*, *Prevotella*, *Veillonella*, *Campylobacter* and *Neisseria* in both 16 S rRNA gene amplicon and metaproteomics results (Fig. 2a). However, the relative abundance distributions of bacterial genera did not show any correlation between the two methods which probably results from relatively higher/lower expression of proteins in some bacterial genera in saliva as expected (Fig. 2b). We calculated genus, family, and phylum level overlap between the methods. The overlap at genus level was 12.4% which increased to 19.7% at family level and 46.2% at phylum level (Fig. 2e). All shared genera, families and phyla are shown in Supplementary Fig. 1. The amplicon sequencing detected much more bacterial taxa at all three levels than metaproteomics profiling. This is not surprising because only a subset of bacterial genera (as described in the Methods) were used to generate a custom-based reference protein database to identify proteins. In addition, we observed a larger fluctuation of bacterial relative abundances based on 16 S rRNA gene amplicon sequencing across samples than that of metaproteomics.

16 S rRNA gene amplicon-based microbiome profiles differentiate study groups

**Fig. 3:**
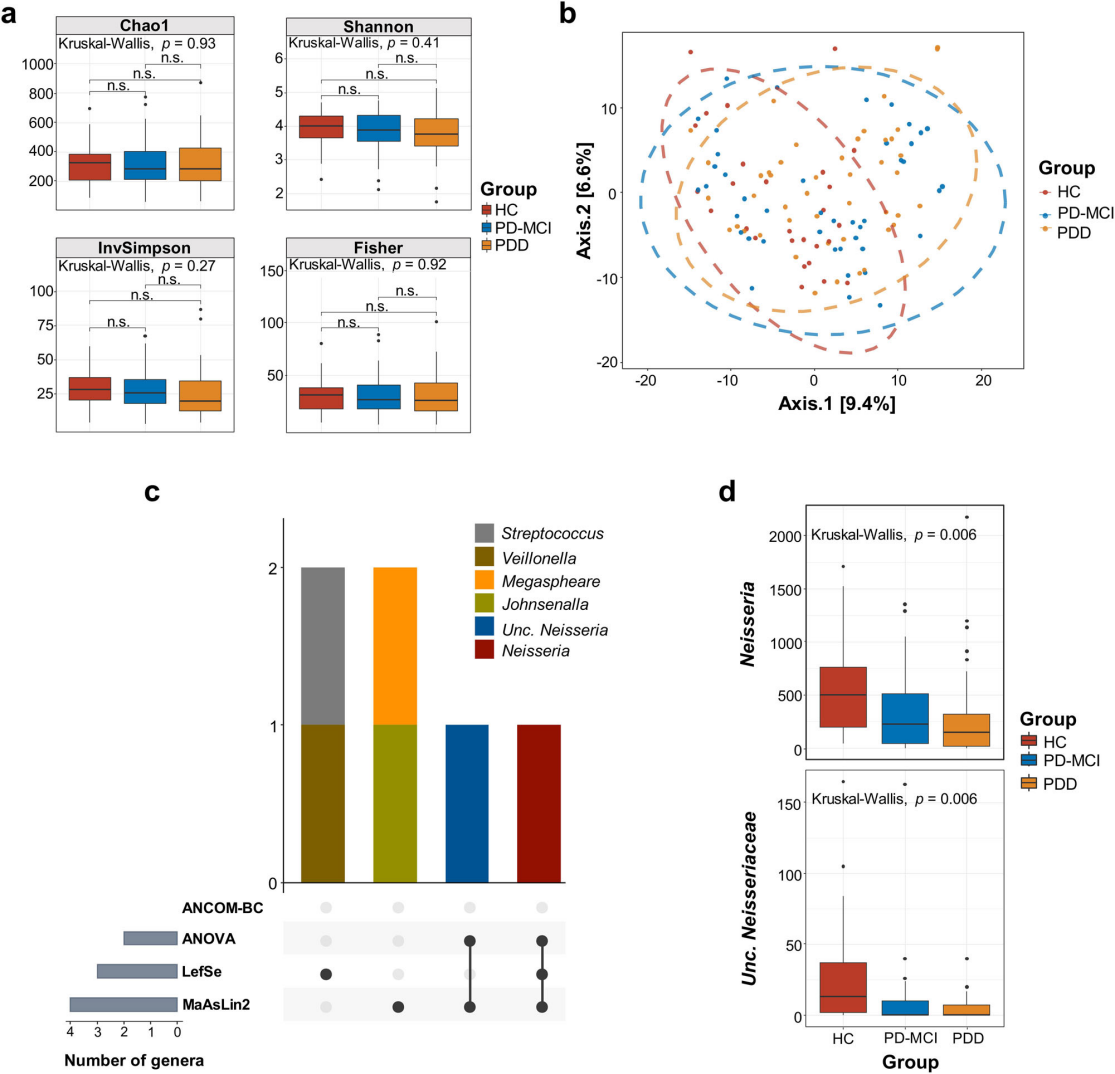
Structural diversity and differential abundance analysis of saliva samples by 16 S rRNA gene amplicon sequencing. **a** Alpha diversity (Chao1, Shannon, InvSimpson, Fisher) comparisons of salivary microbiota samples between study groups. Median estimates compared across study groups using the Kruskal-Wallis test. Boxes represent the interquartile range, lines indicate medians, and whiskers indicate the range. n.s: not significant. **b** Beta diversity comparisons of saliva samples between study groups. PCoA was calculated using Aitchison distance. The ellipses represent a 95% confidence level. Color is indicative of the study group. **c** Number of differentially abundant genera across 4 microbiome differential abundance methods, namely ANCOM-BC, ANOVA, LefSe, MaAsLin2.Vertical bars represent the number of differentially abundant genera shared between the methods highlighted with connected dots in the lower panel. The horizontal bars in the lower panel indicate the total number of differentially abundant genera detected by each method. **d** Abundance distribution of differentially abundant genera detected by at least two methods across study groups. Median estimates compared across study groups using the Kruskal-Wallis test. Boxes represent the interquartile range, lines indicate medians, and whiskers indicate the range. *p*-values represent the overall FDR-corrected *p*-values.

We calculated alpha and beta diversity metrics for saliva samples based on 16 S rRNA gene amplicon sequencing data. There were no significant differences in alpha diversity indices (Chao, Shannon, InvSimpson and Fisher) between study groups (Fig. 3a). On the other hand, beta diversity analyses showed significant differences between study groups (Fig. 3b). We generated a beta-diversity ordination using the Aitchison distance and tested if the samples cluster beyond expected by a chance while adjusting for the confounding effects of age, sex, and education. The results showed a significant difference between the study groups (PERMANOVA, *R*^2^ = 0.021, *adj.p* = 0.027). To strengthen the conclusion, we also we used the Bray-Curtis and Jaccard distance for 16 S rRNA gene amplicon sequencing results to test differences between study groups, which also showed a significant separation between the three groups (Bray-Curtis, PERMANOVA, *R*^2^ = 0.024, *adj.p* = 0.027; Jaccard, PERMANOVA, *R*^2^ = 0.021, *adj.p* = 0.026) (Supplementary Fig. 2).

To determine which microbial taxa were significantly associated with CI, we performed differential abundance using three different tools, namely LEfSe, MaAsLin2, ANCOM-BC and ANOVA (Fig. 3c). Because the correct identification of differentially abundant microbial taxa between experimental conditions varies between different methods^17^, we used three different methods and sought consensus between at least two of these methos in detecting differential abundance. ANOVA-based differential abundance analysis showed a significant decrease in the abundance of *Neisseria* genus and unclassified *Neisseriacceae* with the progression of CI (Fig. 3d) while LEfSe-based analysis not only showed a decrease in *Neisseria* genus but also significant increase in the abundance of *Streptococcus* and *Veillonella* genera with the progression of CI. The decrease in the abundance of *Neisseria* was further supported by MaAsLin2 analysis while ANCOM-BC did not detect any significant difference between study groups. We further investigated potential correlations among bacterial genera and with the covariates MMSE, CDR, UPDRSII. We determined a significant positive correlation between *Neisseria* and MMSE (*p* = 0.003, Spearman’s ρ = 0.268) and a significant negative correlation between *Neisseria* and CDR score (*p* = 0.004, Spearman’s ρ = -0.27) (Supplementary Fig. 3). We have also found a negative correlation of *Lactobacillus* and *Ligilactobacillus* with *Neisseria* (*p* = 0.003, Spearman’s ρ = -0.27 and *p* = 0.03, Spearman’s ρ = -0.20, respectively) (Supplementary Fig. 3).

Metaproteomic profiling identifies human and microbial proteins in saliva

We applied metaproteomics to determine the functional characteristics of the saliva and quantified a total of 29,054 unique peptides and 9379 protein groups in 115 samples. Filtering protein groups that were not identified by at least 2 unique peptide sequences yielded 4253 proteins across our samples. Next, we removed protein groups that were not taxonomically assigned as *Eukaryota* or *Bacteria* which reduced the number of proteins to 3354. Finally, proteins that were not detected in all samples were filtered out to control the batch effect and only the most robustly quantified 537 proteins were used for downstream analyses.

**Fig. 4:**
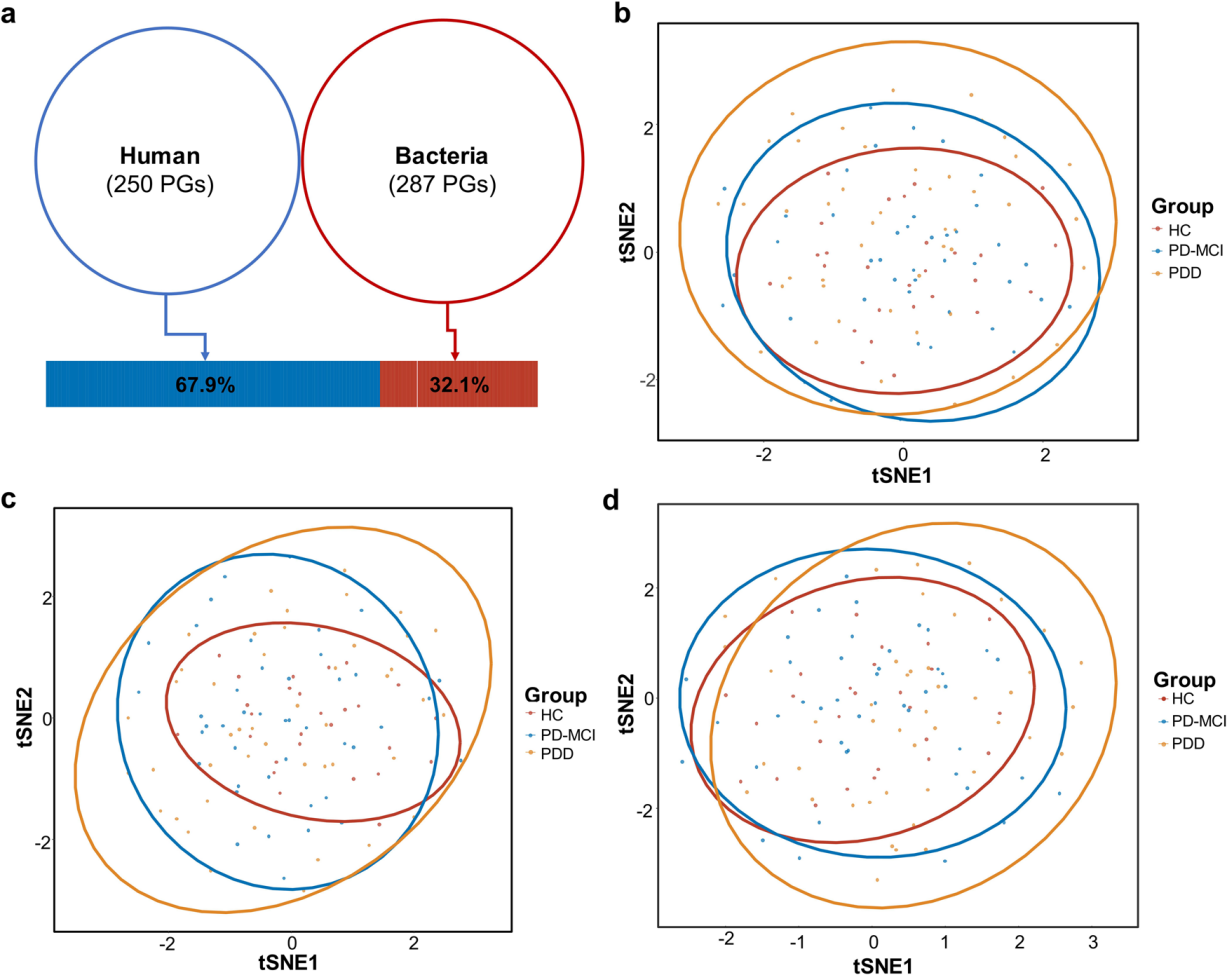
Metaproteome landscape of both human and microbiota protein groups in saliva samples. **a** Distribution of quantified human and microbial protein groups. Venn diagram indicates the numbers of quantified protein groups, while the bar graph shows the total intensity of human or microbial proteins. **b** tSNE plot of bacterial protein groups quantified in saliva samples (PERMANOVA, *R*^2^ = 0.20, *adj.p* = 0.089). **c** tSNE plot of human protein groups quantified in saliva samples (PERMANOVA, *R*^2^ = 0.21, *adj.p* = 0.106). **d** t-Distributed Stochastic Neighbor Embedding (t-SNE) plot of all proteins quantified in saliva samples (PERMANOVA, *R*^2^ = 0.026, *adj.p* = *0.025*). Beta diversity comparisons were performed using Aitchison distance. The ellipses represent a 95% confidence level. Color is indicative of the study group.

Among 537 protein groups quantified, 287 (53.5%) were from salivary microorganisms while 250 (46.5%) protein groups were from human proteome origin. Human protein groups constituted 67.9% of the total protein intensities measured in the salivary samples while microbial protein groups constituted 32.1% of total protein intensities (Fig. 4a) which shows that human proteins are more abundant in saliva and consistent with previous studies^18^. We tested if the ratio between human and microbial protein groups changes with the progression of the CI and detected no significant change (Supplementary Fig. 4). We also performed beta diversity analysis based on human and microbial proteins separately to evaluate if there is a significant association between human or microbial protein group composition and CI. The results did not show any compositional difference in microbial or human protein groups profiles across study groups (Fig. 4b, c). However, beta diversity analysis based on all detected proteins showed significant separation between groups (PERMANOVA, *R*^2^ = 0.026, *p* = 0.025); although when adjusted for all potential confounders, the difference was attenuated (PERMANOVA, *R*^2^ = 0.019, *adj.p* = 0.082) (Fig. 4d).

**Fig. 5:**
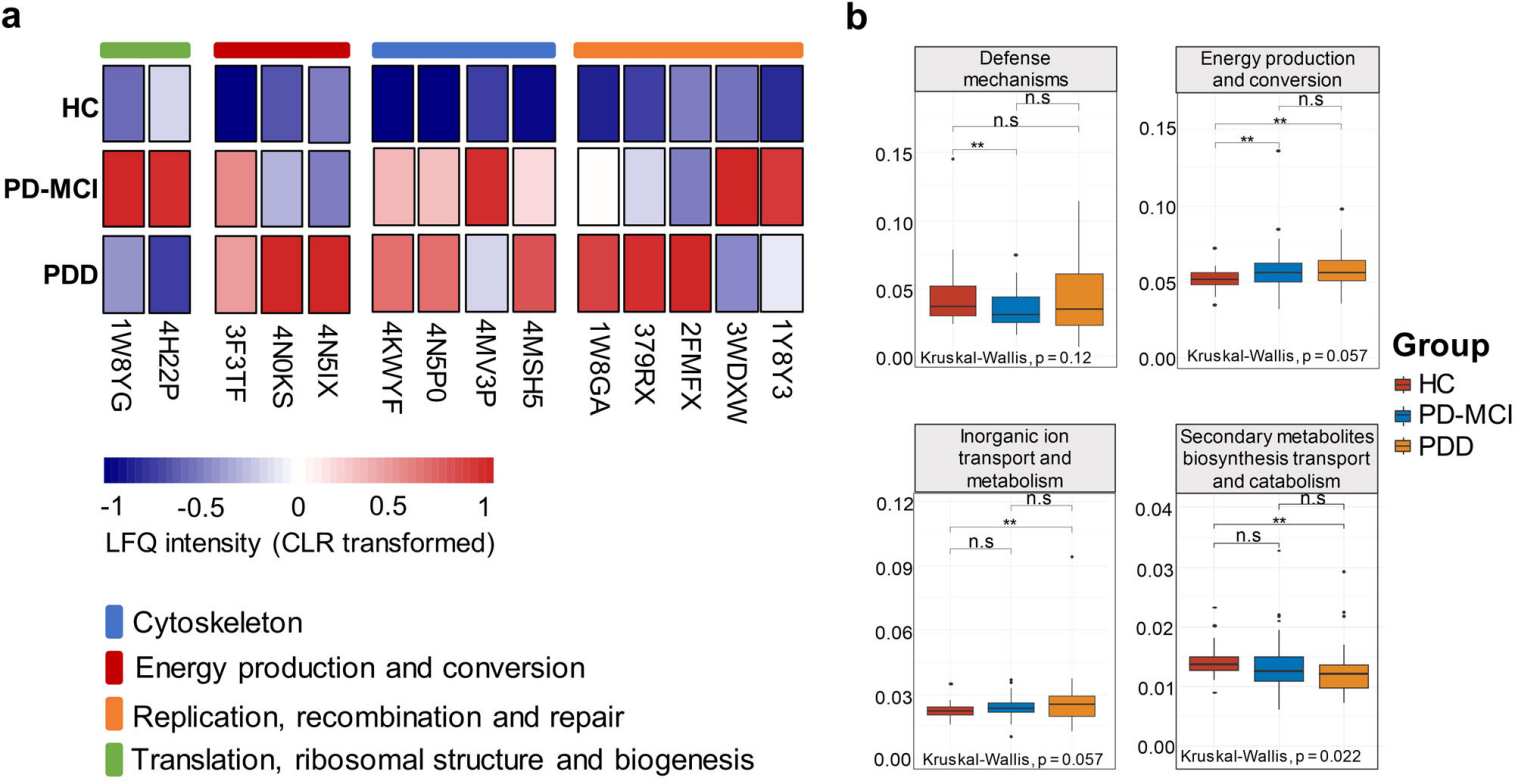
Functional categories of differentially abundant proteins in the saliva samples. **a** Heatmap of differentially abundant OGs between the study groups. Representative OG categories are shown, and the colors indicate the average label-free quantification (LFQ) intensity for each study group. Each row corresponds to an OG with the OG id. CLR: Centered log ratio. **b** LFQ intensity of functional category V (defense mechanism), category C (energy production and conversion), category P (inorganic ion transport and metabolism) and category Q (secondary metabolites biosynthesis transport and catabolism) in saliva samples across the study groups. Median estimates compared across study groups using the Kruskal-Wallis test. Boxes represent the interquartile range, lines indicate medians, and whiskers indicate the range. n.s: not significant, *p < 0.1, **p < 0.05.

In order to assess changes in the functional profile of samples, we annotated protein groups using Prophane and applied differential abundance tests both at OG and functional category levels. We have obtained 371 OGs from 24 functional categories and 25 differentially abundant OGs between study groups. At the OG level analysis, functions related to the replication, recombination, and repair (5 OGs in category L), cytoskeleton (4 OGs in category Z), energy production and conversion (3 OGs in category C), translation, ribosomal structure, and biogenesis (2 OGs in category J) were among the most significantly different categories between study groups. 20 of differentially abundant OGs were significantly increased with the progression of CI while 5 OGs showed an increase from HC to MCI and a decrease from MCI to PDD (Fig. 5a). The differential abundance results for all OGs and functional categories are shown in Supplementary Fig. 5 and Supplementary Fig. 6, respectively.

At the functional category level, energy production and conversion and inorganic ion transport and metabolism categories were significantly increased with the progression of CI while defense mechanisms and secondary metabolites biosynthesis, transport and catabolism displayed a decreased abundance (Fig. 5b).

### Multi-omics factor analysis (MOFA)

**Fig. 6:**
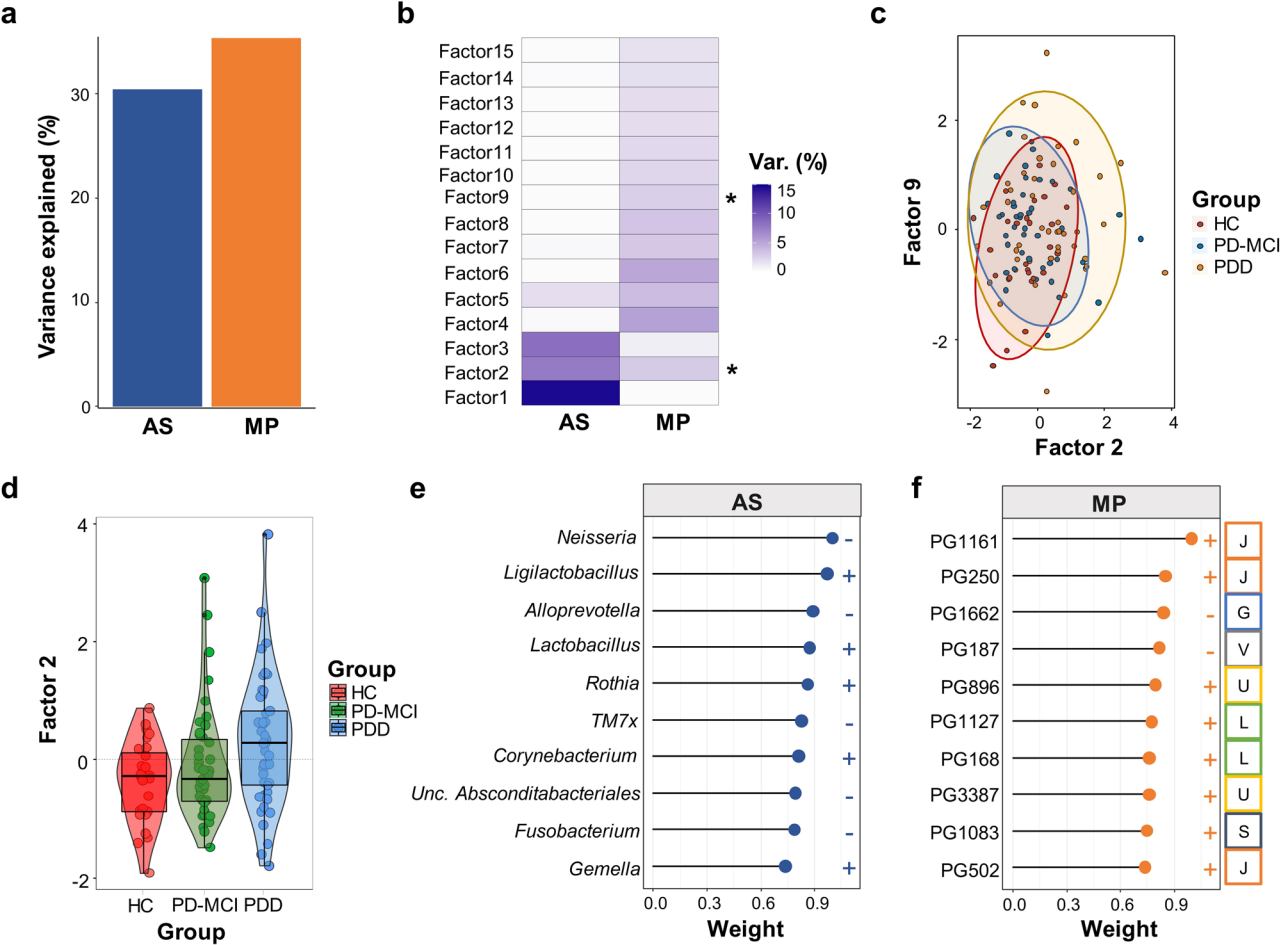
Multi-omics factor analysis (MOFA) of metaproteogenomics dataset. **a** Fraction of total variance explained by type of measurement (view). **b** Fraction of total variance explained by latent factors (LFs) 1–15. Stars indicate significant association of the factor with study groups. **c** Scatter plot of latent Factor 2 (x axis) and latent Factor 9 (y axis). Dots and ellipses are colored according to their group assignment. **d** Box plot of latent Factor 2 values grouped and colored by CI status. **e** Lollipop plot shows top ranking bacterial genera in latent Factor 2. **f** Lollipop plot shows top ranking salivary PGs in latent Factor 2. Human protein groups are colored orange, bacteria protein groups are colored blue.

We applied MOFA to integrate amplicon sequencing and metaproteome results. The fitted model explained 30.1% and 34.4% of the variance in 16 S rRNA sequencing and metaproteomics datasets, respectively (Fig. 6a) with latent 15 factors (Fig. 6b). We examined potential correlations between factors and covariates. We observed that only two of these factors (Factor 2 and Factor 9) were significantly associated with CI in PD and included both bacterial genera and protein features (Fig. 6c). Factor 9 has been found to be correlated with Age covariate along with CI stages (Supplementary Fig. 7). Thus, we focused on Factor 2 because it was the only factor associated with the progression of CI (*R*^2^ = 0.064, *p* = 0.003) and identified the contributing features of this factor. Factor 2 values positively correlated with the progression of CI (Fig. 6d). Amplicon sequencing based microbiome component of the Factor 2 revealed a decreased abundance of the *Neisseria, Alloprevotella, TM7x, Unclassified Absconditabacteriales* and *Fusobacterium* and increased abundance of *Ligilactobacillus, Lactobacillus, Rothia, Corynebacterium* and *Gemella* in association with the progression of CI in PD. Metaproteome component of Factor 2 showed increased abundance of proteins associated with translation, ribosomal structure and biogenesis (category J), intracellular trafficking, secretion, and vesicular transport (category U), replication, recombination and repair (category L) and unknown function (category S) and a decreased abundance of proteins associated with carbohydrate transport and metabolism (category G) and defense mechanisms (category V).

### Comparisons with PD patients with normal cognition (PDNC)

We calculated alpha and beta diversity metrics for saliva samples based on 16 S rRNA gene amplicon sequencing data including 123 samples from our study (subjects (HC, *n* = 27; PD-MCI, *n* = 45; PDD, *n* = 43; PDNC, *n* = 8) and 40 samples (HC, *n* = 20; PDNC, *n* = 20) from a published study)^15^. Alpha diversity comparisons showed no significant difference between sample groups (Supplementary Fig. 8a). On the other hand, the results showed that beta diversity of saliva microbiota is dissimilar across the range of cognitive impairment as compared with PDNC and HC groups (PERMANOVA, *R*^2^ = 0.036, *p* = 0.001) (Supplementary Fig. 8b). In addition, we performed differential abundance analysis and confirmed the association of same bacterial taxa with PD groups with CI (PD-MCI and PDD) when compared to PDNC and HC groups (Supplementary Fig. 9).

Furthermore, we repeated MOFA analysis adding 8 PDNC samples as the fourth sample group to our cohort. We examined potential correlations between factors and covariates and determined the association of Factor 2 primarily with CI in PD and Disease Duration (PD-MCI, *p* = 0.01; PDD, *p* = 0.02; PDNC, *p* = 0.44, Disease Duration, *p* = 0.0002) (Supplementary Fig. 10a). Factor 2 values negatively correlated with the progression of CI (Supplementary Fig. 10b). Amplicon sequencing based microbiome component of the Factor 2 revealed a decreased abundance of the *Neisseria, Alloprevotella, TM7x* and *Fusobacterium* and increased abundance of *Ligilactobacillus, Lactobacillus* and *Rothia* in association with the progression of CI in PD which were consistent with our previous analysis results. Metaproteome component of Factor 2 supported the association of 2 PGs (pyruvate, phosphate dikinase (PPDK)) and bactericidal permeability-increasing protein (BPI) identified in our previous analysis without PDNC group.

## Discussion

In this study, we characterized the compositional dynamics of saliva of PD patients across a continuum of CI (PD-MCI and PDD) as compared with HC using both 16 S rRNA gene amplicon sequencing and metaproteomics profiling. We identified the discriminatory key signatures in saliva composition related to CI stages of PD by applying an integrative metaproteogenomics approach. Employing an integrative analysis of saliva metagenomics and metaproteomics, we demonstrated that a shift in salivary microbiome and protein translation machinery and defense mechanism related changes in human proteome is associated with the CI progression in PD.

Both metaproteome and microbiota profiles indicated *Streptococcus, Prevotella, Veillonella, Fusobacterium* and *Neisseria* as the most abundant bacterial genera in the study cohort which is in agreement with a previous study comparing salivary microbiota of healthy controls and PD patients^15^. The amplicon sequencing detected much more bacterial genera, families, and phyla than metaproteomics profiling. This is not surprising because only a subset of bacterial genera (as described in the Methods) were used to generate a custom-based reference protein database to identify proteins.

Although alpha diversity in amplicon-based microbiota showed no significant differences between study groups beta diversity comparisons of saliva samples significantly differentiated CI stages, suggesting salivary bacterial community restructures by the progression of CI. Results of four differential abundance methods showed in consensus a significant decrease of *Neisseria* genus with the progression of CI, which aligned well with the previously published reports^19,20^. Here, we further showed the continuous decrease of *Neisseria* with the progression of CI in PD.

The metaproteome profile of saliva samples was composed of a balanced number of human and bacterial protein groups. On the other hand, human proteins constituted much higher percentage of total protein intensities measured in the saliva samples, as observed in previous studies^18^. Differential abundance analyses of metaproteome profiles determined marked functional alterations associated with CI. Particularly, the functions related to cytoskeleton and translation, ribosomal structure and biogenesis, defense mechanisms and energy production and conversion were among the most significantly altered functions between study groups.

The taxa *Neisseria* deserves special attention among others as it is implicated in prevention of oral diseases due to its beneficial abilities such as metabolizing low-pH products into weak acids. On the other hand, consistently reported high abundance of *Lactobacillaceae* members in the oral cavity of PD patients may have negative effects due to their ability to reduce secretion of neuroprotective hormones such as ghrelin^21^. The decrease in *Neisseria* accompanied with the increased abundance of *Lactobacillaceae* family has been also reported in a recent study on PD^16^. Our results suggest a continuity for this compositional shift across CI spectrum in PD. Moreover, we have detected a decrease in PPDK enzyme, as another signature. PPDK is known as one of key enzymes in gluconeogenesis^22^ and related to lactic acid production^23^. Furthermore, we detected BPI as another signature protein group, which is involved in the defense of host against bacterial pathogens and considered as a microbial translocation markers^24^. Elevated serum endotoxin levels have been previously reported in PD patients, which indicate greater bacterial translocation, particularly for the subgroup with high risk for early dementia^25^. Thus, our observation of decreased BPI abundance in saliva suggests a potential LPS-BPI imbalance in PD patients which worsens with the CI progression.

In conclusion, our study presents a comprehensive overview of significant key changes in saliva composition that parallel the progression of CI in PD and suggests potential non-invasive biomarker candidates for predicting CI in PD by integrating amplicon sequencing and metaproteomics. This approach paves the way for developing non-invasive biomarker strategies to predict the risk of CI progression in PD.

The conclusions drawn from the data we presented in this manuscript should be interpreted under the following considerations.

In spite of the valuable insights gained from our microbiome study, it is essential to acknowledge the limitations that may impact the interpretation and generalizability of our findings. The absence of adequate controls for confounding variables, such as dietary habits (although we confirmed that the participants did not have strict dietary habits) and medication usage may introduce biases and hinder our ability to establish direct causal relationships between the microbiome and cognitive impairment in PD. Another limitation is the poor representation of PD patients without cognitive impairment (PDNC) in our cohort as a comparison group. Even though we attempted to rectify this limitation in our study design by adding published data generated from PDNC group we were unable to pinpoint a dataset including both metaproteomics and amplicon sequencing from the same saliva samples obtained from PDNC group. Thus, even though the microbial taxa found to be associated with CI in our study is robust we have less confidence in the saliva proteins linked with the confidence impairment due to the poor representation of PDNC group in our cohort. Regardless, these findings should be validated in a prospective longitudinal study in the future. Despite these limitations, however, our study contributes valuable preliminary insights into associations of putative saliva biomarkers with CI in PD. Future research endeavors should aim to address these confounding factors rigorously.

## Methods

### Study subjects and clinical characteristics

The study was approved by the ethics committee of the Istanbul Medipol University with authorization number 10840098-604.01.01-E.3958, and informed consent was obtained from all participants. If the patient has progressed into dementia stage and was not able to make independent assessment of the consent form, consent of the patient’s immediate family member (spouse, children, or the caregiver) was obtained. A total of 115 subjects (HC, *n* = 27; PD-MCI, *n* = 45; PDD, *n* = 43) within the age range of 50-75 were recruited at two tertiary training hospitals including the Medipol Training and Research Hospital in the neurology clinic and Bakirkoy Research and Training Hospital for Psychiatric and Neurological Diseases. The subjects in the HC group were recruited largely from the family members of the patients and also some family members of the hospital employees and of the students, otherwise from the individuals who responded to the advertisement of the clinical study. All control group participants were required to take formal neuropsychological testing and assessed by the clinician on their cognitive capacity, and by the exclusion criteria of the study. Subjects with previous head trauma, stroke, or exposure to toxic substances, substance abuse, history of antibiotic or probiotic use within last 1-month, chronic severe diseases (diabetes, cancer, kidney failure, etc.), autoimmune diseases, smokers, pregnancy, and those with symptoms suggestive of Parkinson’s plus syndromes were excluded from the study. Clinical and demographic information, including age, sex, years of education were collected at clinic visits. The patients were examined by experienced neurologists and the diagnosis of PD was made within the framework of the “United Kingdom Parkinson’s Disease Society Brain Bank” criteria. Hoehn-Yahr Stages Parkinson’s Staging Scale was used to determine the stage of the disease and The Movement Disorder Society’s diagnostic criteria for Parkinson’s Disease Dementia criteria were used for dementia evaluation according to Emre et al.^26^ The diagnosis of MCI was made within the framework of the criteria defined by Litvan et al.^27^ according to level II criteria (comprehensive cognitive assessment based on the MDS task force diagnostic criteria (neuropsychological testing that includes two tests within each of the five cognitive domains).

### Sample preparation for 16 S rRNA gene amplicon gene sequencing

Unstimulated saliva samples were divided into two equal aliquots of 500 ml and used for DNA and protein extractions separately. Microbial DNA extraction from saliva samples was performed using DNeasy PowerSoil (Qiagen, Hilden, Germany) with modifications as described before^28^. In brief, 250 ml saliva sample was centrifuged at 10,000 x g for 5 min, supernatant discarded, and the pellet was resuspended with 400 μl bead beating buffer and transferred to the PowerBead tube. Samples were homogenized by bead-beating using Next Advance Bullet Blender (30 s at level 4, 30 s incubation on ice and 30 s at level 4). After bead-beating step, the manufacturer’s protocol was followed without any modification. The V3-V4 regions of 16 S rRNA gene were amplified using the universal bacterial primers (F-5′-CCTACGGGNGGCWGCAG-3′ and R-5′-GACTACHVGGGTATCTAATCC-3′). Next, amplicon libraries were prepared by following Illumina’s 16 S rRNA metagenomic sequencing library preparation protocol and sequenced using a MiSeq platform and 2 × 250 paired end kit. Amplicon sequencing libraries prepared from a total of 115 gDNA samples were sequenced along with DNA extraction negative control and a no-template PCR control per run.

### Sample preparation for metaproteomics

For protein extraction, 250 ml saliva sample was centrifuged at 10,000 x g for 5 min. Discarding the supernatant, the pellet was resuspended in 250 μl bead beating buffer and transferred to the BeadBug™ prefilled tubes, 2.0 ml containing 1.0 mm Zirconium beads. Samples were homogenized by bead-beating using Next Advance Bullet Blender (30 s at level 4, 30 s incubation on ice and 30 s at level 4). After bead-beating step, samples were incubated at 100 °C for 10 min under constant shaking (600 rpm), followed by 4 °C incubation for an hour and centrifugation at 16,000 × g for 10 min. The supernatants were finally transferred to a clean 1.5 ml microcentrifuge tube. The total protein concentrations were measured using the Qubit protein assay kit. 50 µg protein (eluted in 30 µl) was used in the downstream analyses for each sample. Tryptic peptides were generated using the Filter Aided Sample Preparation Protocol (FASP) kit (Expedeon, San Diego, USA) according to the manufacturer’s protocol. The peptides were dissolved in 0.1 percent formic acid and diluted to 100 ng/μl before injecting to the liquid chromatography tandem-mass spectrometry (the ACQUITY UPLC M-Class coupled to a SYNAPT G2-Si high-definition mass spectrometer (Waters, Milford, CT)). The LC-MS/MS analysis was performed according to a previously published protocol^29^.

### Analysis of 16 S rRNA gene amplicon sequencing data

Raw 16 S rRNA gene amplicon sequencing data were analyzed using the Nephele platform (v.1.6, http://nephele.niaid.nih.gov)^30^ using default parameters (QC: minlen: 30, req_qual: 12, overlap: 3, trail_qual: 3, run_flash2_merge: True, f2_min_overlap: 10, run_flash2_merge: True, error_rate: 0.1, window_size: 4; DADA2: maxEE: 5, ref_db: sv138.1, taxmethod: rdp, trimleft_fwd: 0, truncLen_fwd: 0, truncQ: 4) and SILVA v.138.1 database^31^. The contaminant sequences were identified and removed using the *decontam* package^32^ based on negative control samples. Only ASVs present in at least 2 samples were included in the downstream analyses. Samples were rarefied to minimum sampling depth (4,821 reads) before performing alpha diversity analyses and CLR transformed before beta diversity analyses. Diversity analyses were performed using QIIME2^33^ and phyloseq^34^. MaAsLin2^35^, LEfSe^36^, ANCOM-BC^37^ and ANOVA tests were used to determine differential abundances between groups, controlling covariates when possible. The R package ggplot2^38^ was used for visualization of the results.

### Analysis of metaproteomics data

For metaproteomics data analysis, firstly, a custom protein database based on 16 S rRNA amplicon sequencing results was built. Briefly, we determined the top 20 most abundant bacterial genera across study groups. We obtained all species-level genome bins that belonged to these genera from a recently published comprehensive study on human oral microbiome^39^. Next, we added all human proteins from UniProt database^40^ to the predicted bacterial proteins of these genome bins which produced a final protein database of 1,165,589 protein sequences. Progenesis-QI (Waters) software was used to identify and quantify the protein groups. Protein groups identified by at least 2 unique peptide sequences, taxonomically assigned to *Eukaryota* or Bacteria kingdoms and present in all samples were used for downstream analyses. Also, to reduce batch effects on metaproteomics results, we used MMUPHin^41^ to adjust protein abundances before further processing. Diversity analyses were performed using phyloseq. We employed Prophane^42^ tool with default parameters to annotate taxonomy and functions of the detected proteins. All identified protein groups from each sample were classified into main metabolic pathways using the orthologous groups (OG) classification against the EggNog database^43^. ANOVA was used to examine potential associations at both the protein OGs and functional categories levels with CI stages.

### Multi-omics factor analysis

Multi-Omics Factor Analysis (MOFA)^44^ was employed to integrate 16 S rRNA amplicon sequencing and metaproteomics datasets as data modalities with matching samples. ASV table was collapsed to the genus level and both datasets were centered log ratio (CLR) transformed to reduce compositionality bias before generating the MOFA model. Data and training options were set to default with 10,000 iterations in ‘slow’ convergence mode to generate 15 factors. Downstream analyses after generation of MOFA model were performed using MOFA+ tool^45^.

### Comparisons with PD patients without cognitive impairment (PDNC)

We used two approaches to verify the reliability of our findings regarding the correlation between our selected features and CI in PD. Firstly, we incorporated data from a previously published study conducted by Fleury et al.^15^ that employed 16 S rRNA gene amplicon sequencing. This study compared 20 PDNC to 20 HC, targeting the V3-V4 region of the 16 S rRNA gene and using the same primer set we used in this study. We followed the same analysis steps as described earlier to reinforce our findings and validate the association of specific bacterial genera with PD-MCI and PDD groups. Secondly, we gathered an additional 8 PDNC samples and analyzed them using metaproteomics and amplicon sequencing techniques.

### Statistical analyses

Statistical analyses were conducted in R 3.6.1. A Kruskal-Wallis test was used for alpha diversity comparisons. Adonis2, an implementation of permutational multivariate analysis of variance (PERMANOVA) from the vegan package with 999 permutations, was used for beta diversity comparisons, with adjustment for potential confounding factors Age, Sex, Education, Disease Duration and HYE. Linear regression analysis was performed to test association of latent factors generated by MOFA, with adjustment for potential confounding factors. A *t*-test was used for continuous variables, namely age and education, while Fisher’s exact test was used for categorical variables. All *p*-values, where appropriate, were adjusted for multiple testing using the Benjamini-Hochberg method. Adjusted *p*-values are denoted by “adj.p” and raw *p*-values are denoted by “*p*” throughout the manuscript. Results of all statistical tests are provided in the Supplementary File 1.

### Reporting summary

Further information on research design is available in the Nature Research Reporting Summary linked to this article.

### Supplementary information

Supplementary file 1

Reporting Summary

## Data Availability

The raw 16 S rRNA gene amplicon sequencing data produced in this study have been deposited in the NCBI Sequence Read Archive database under accession no. PRJNA913682. Metaproteomics data generated and analyzed in this study are available from the corresponding author on reasonable request.

## Code availability

No new codes were generated or used in this study.
