# Supplementary material for: Metaproteogenomic analysis of saliva samples from Parkinson’s disease patients with cognitive impairment
Source: NPJ Biofilms Microbiomes. 2023 Nov 18;9:86. doi: 10.1038/s41522-023-00452-x (PMC10657361; doi:10.1038/s41522-023-00452-x)
Supplement: Supplementary file 1 [file 41522_2023_452_MOESM1_ESM.pdf]

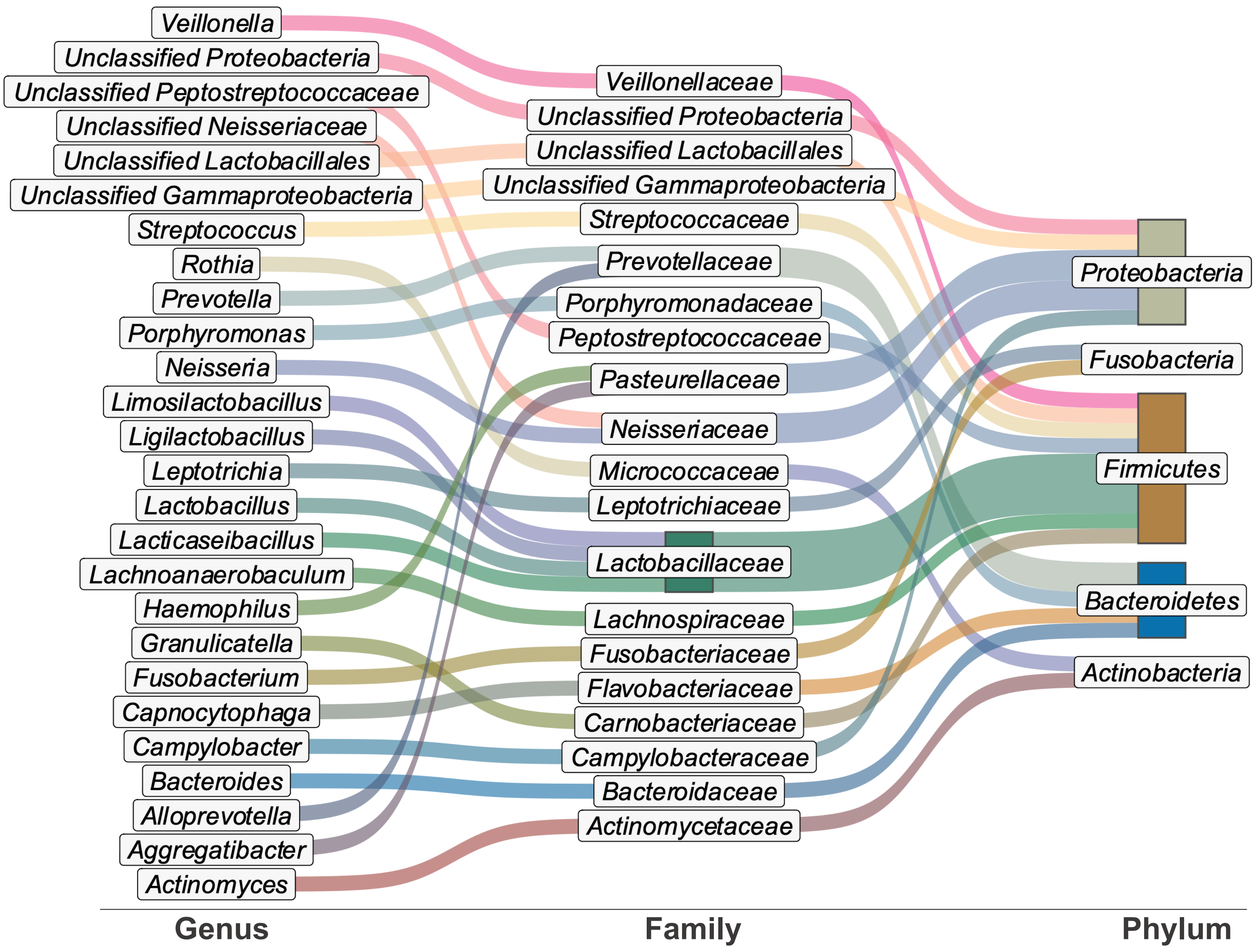


**Supplemental Figure 1.** Sankey plot based on assigned taxonomy showing the shared bacterial taxa between amplicon sequencing and metaproteomics methods at three phylogenetic levels. Taxonomy assignments and comparisons resulted in 26 genera, 20 families and 5 phyla shared between two methods.

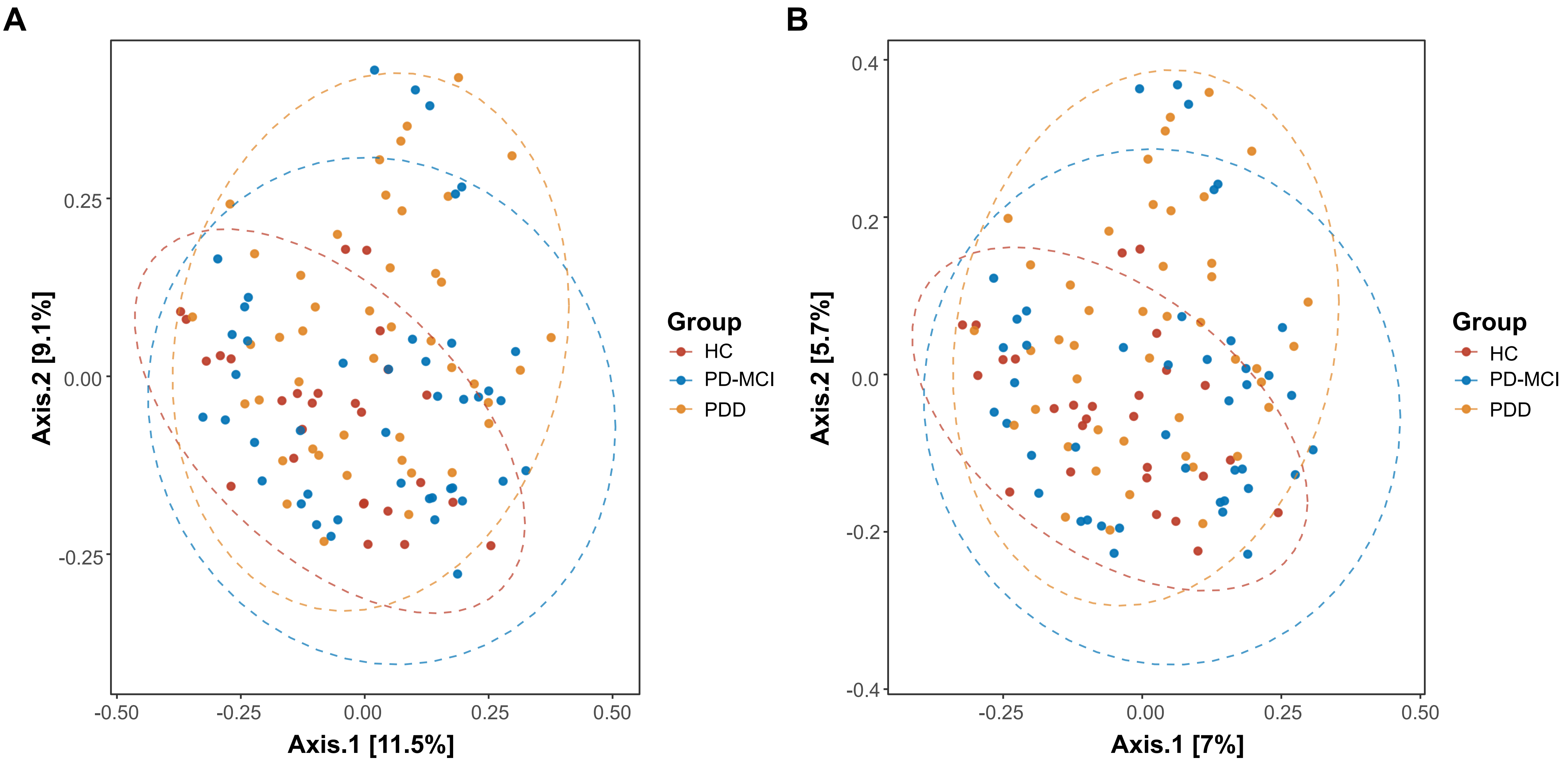


**Supplemental Figure 2.** Beta diversity comparisons of saliva samples between study groups by 16S rRNA gene amplicon sequencing. (A) Bray-Curtis (PERMANOVA, R2 = 0.024, p = 0.021) and (B) Jaccard (PERMANOVA, R2 = 0.021, p = 0.017) distance-based beta diversity comparisons of saliva samples between study groups. PCoA was calculated using Aitchison distance. Ellipses represent an 95% confidence level. Color is indicative of the study group.

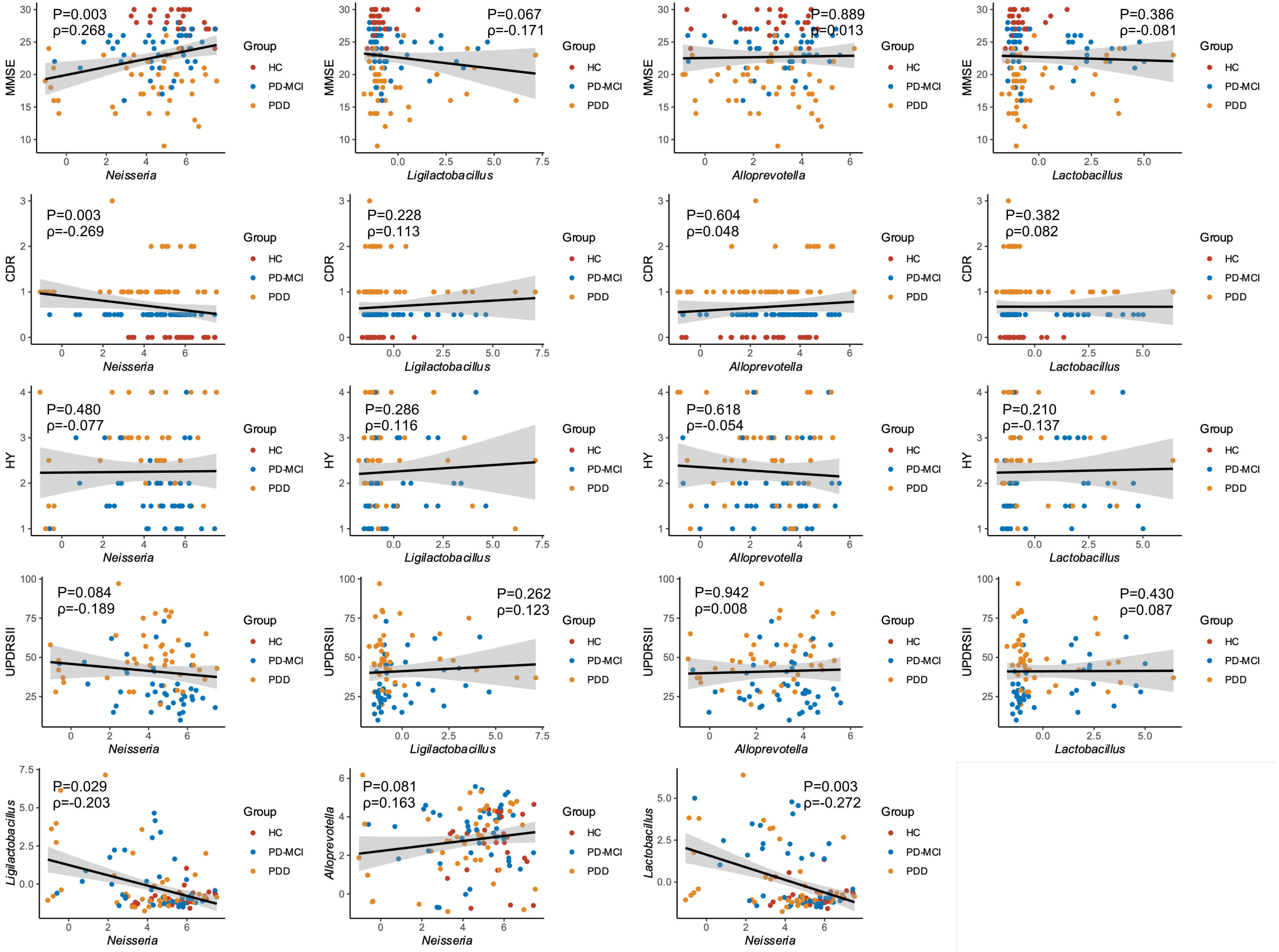


**Supplemental Figure 3.** Correlations of four bacterial genera (*Neisseria, Alloprevotella, Ligilactobacillus and Lactobacillus*) with each other and covariates MMSE, CDR and UPDRSII. *Neisseria* had a significant positive correlation with MMSE (p=0.003, Spearman's ρ=0.268) and a significant negative correlation between with CDR score, *Lactobacillus* and *Ligilactobacillus* (p=0.003, Spearman's ρ=-0.269, p=0.003, Spearman's ρ=-0.272 and p=0.029, Spearman's ρ=-0.203, respectively. Color is indicative of the study group.

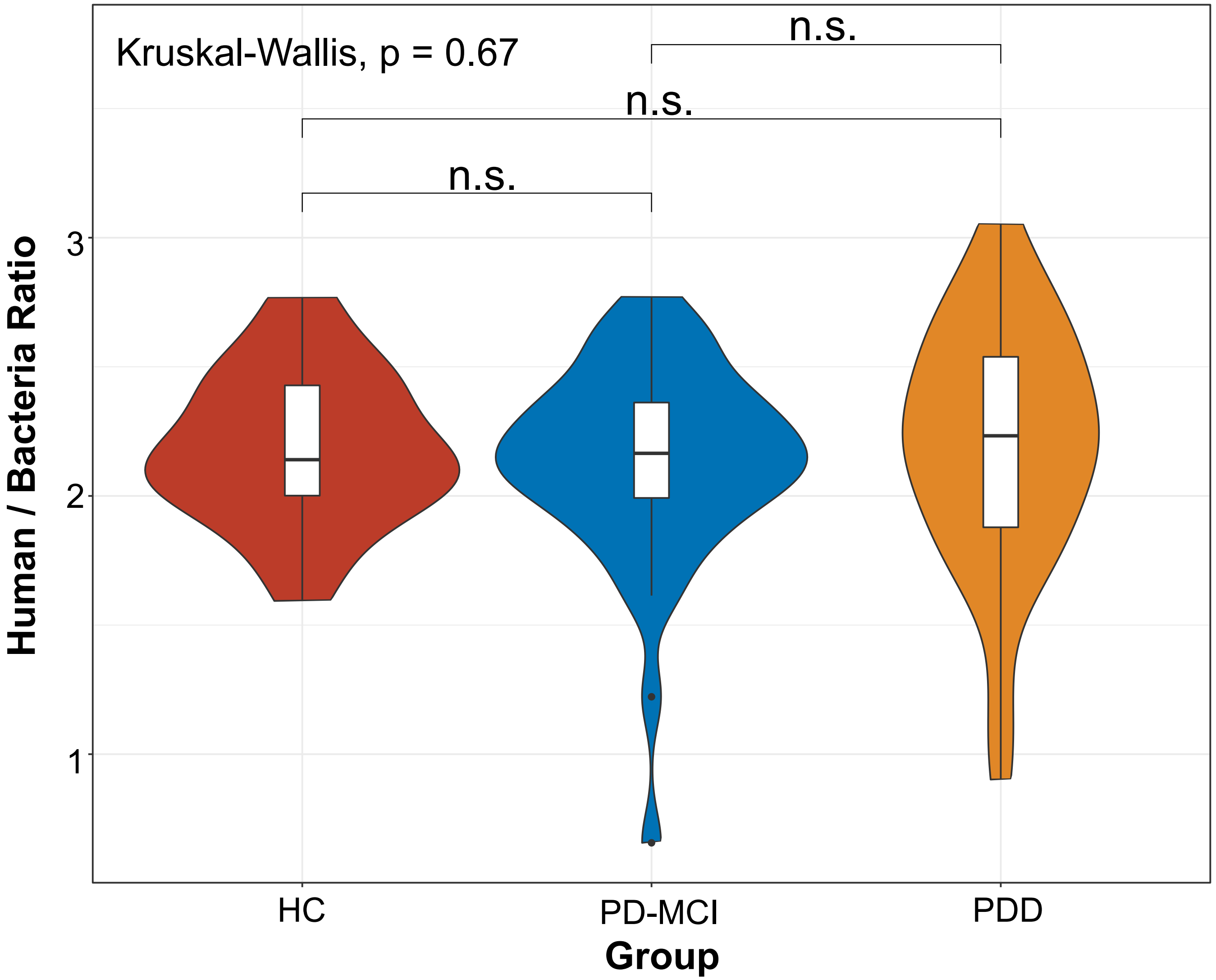


**Supplemental Figure 4.** Human-to-bacteria ratios of total protein intensity. The middle line represents the sample median. Median estimates compared across study groups using the Kruskal-Wallis test. Boxes represent the interquartile range, lines indicate medians, and whiskers indicate the range. p values represent the overall FDR-corrected p values. Color is indicative of the study group. n.s: not significant, *p < 0.1, **p < 0.05.

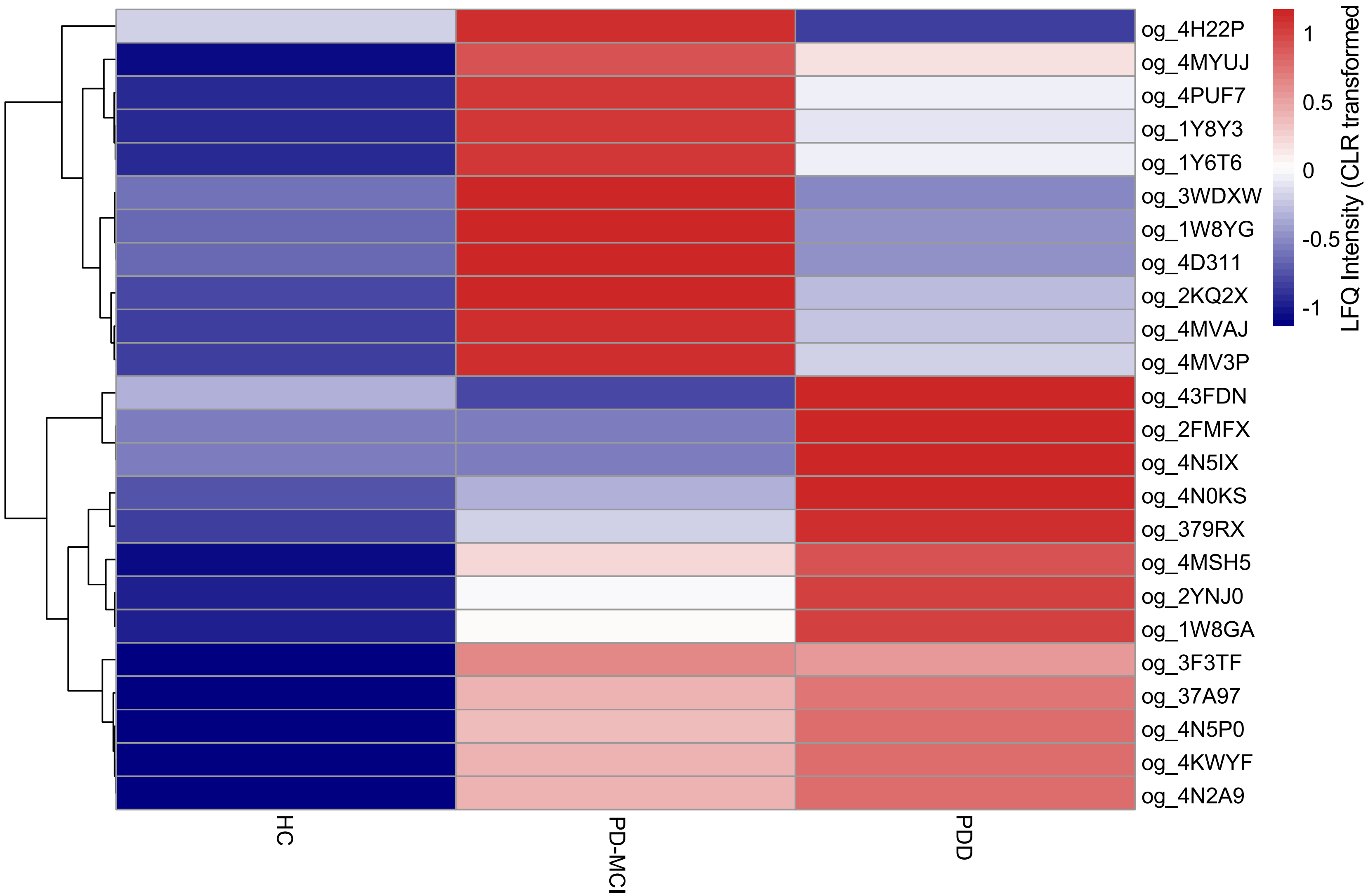


**Supplemental Figure 5.** Heatmap of differentially abundant orthologous groups (OG) between the study groups. The colors indicate the average label-free quantification (LFQ) intensity for each group. Each row corresponds to an OG with the OG id. CLR: Centered log ratio.

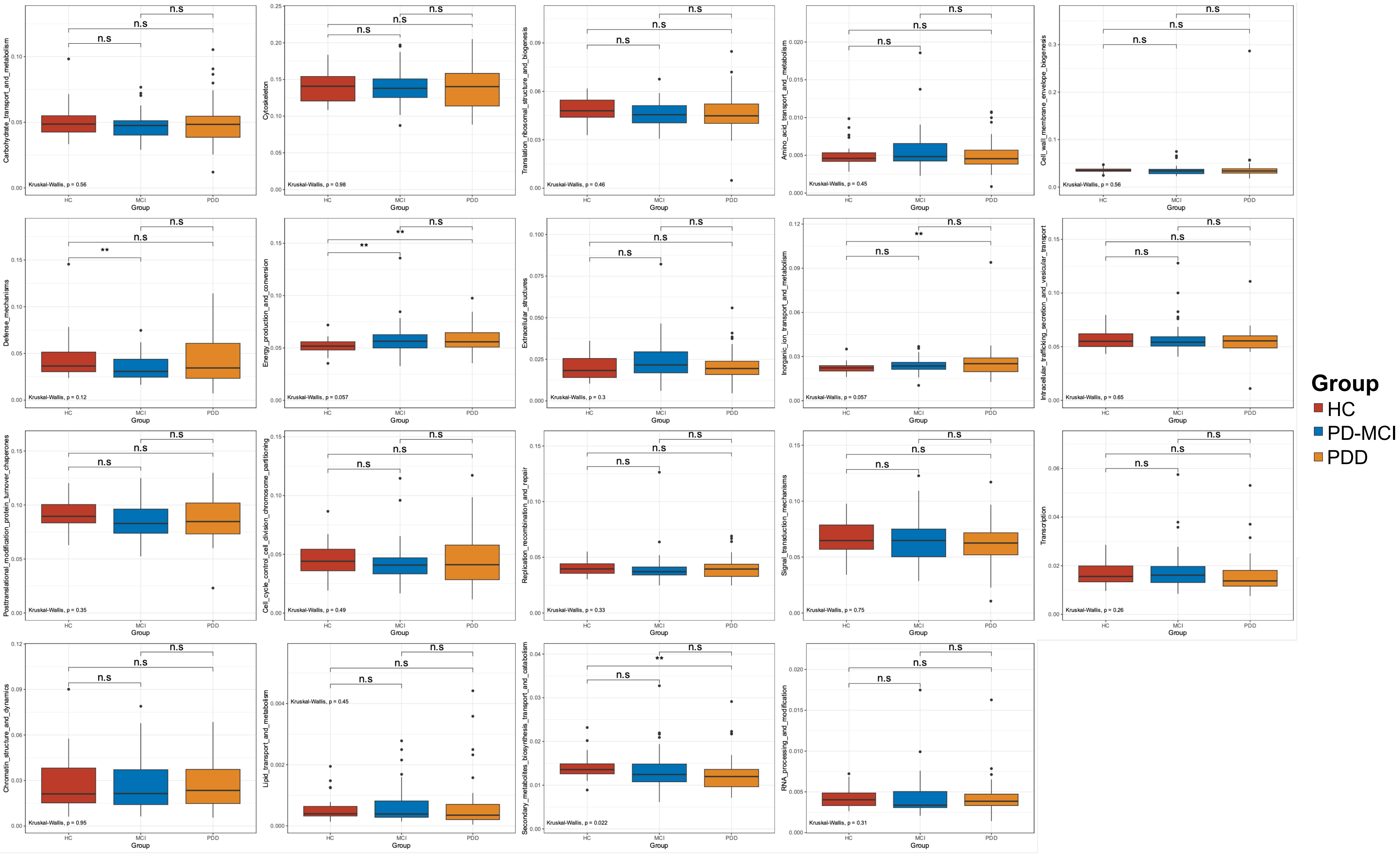


**Supplemental Figure 6.** The differential abundance results for all functional categories. Median estimates compared across study groups using the Kruskal-Wallis test. Boxes represent the interquartile range, lines indicate medians, and whiskers indicate the range. n.s: not significant, *p < 0.1, **p < 0.05. Defense mechanisms, energy production and conversion, inorganic ion transport and metabolism and secondary metabolites biosynthesis transport and catabolism categories were significantly different between the study groups.

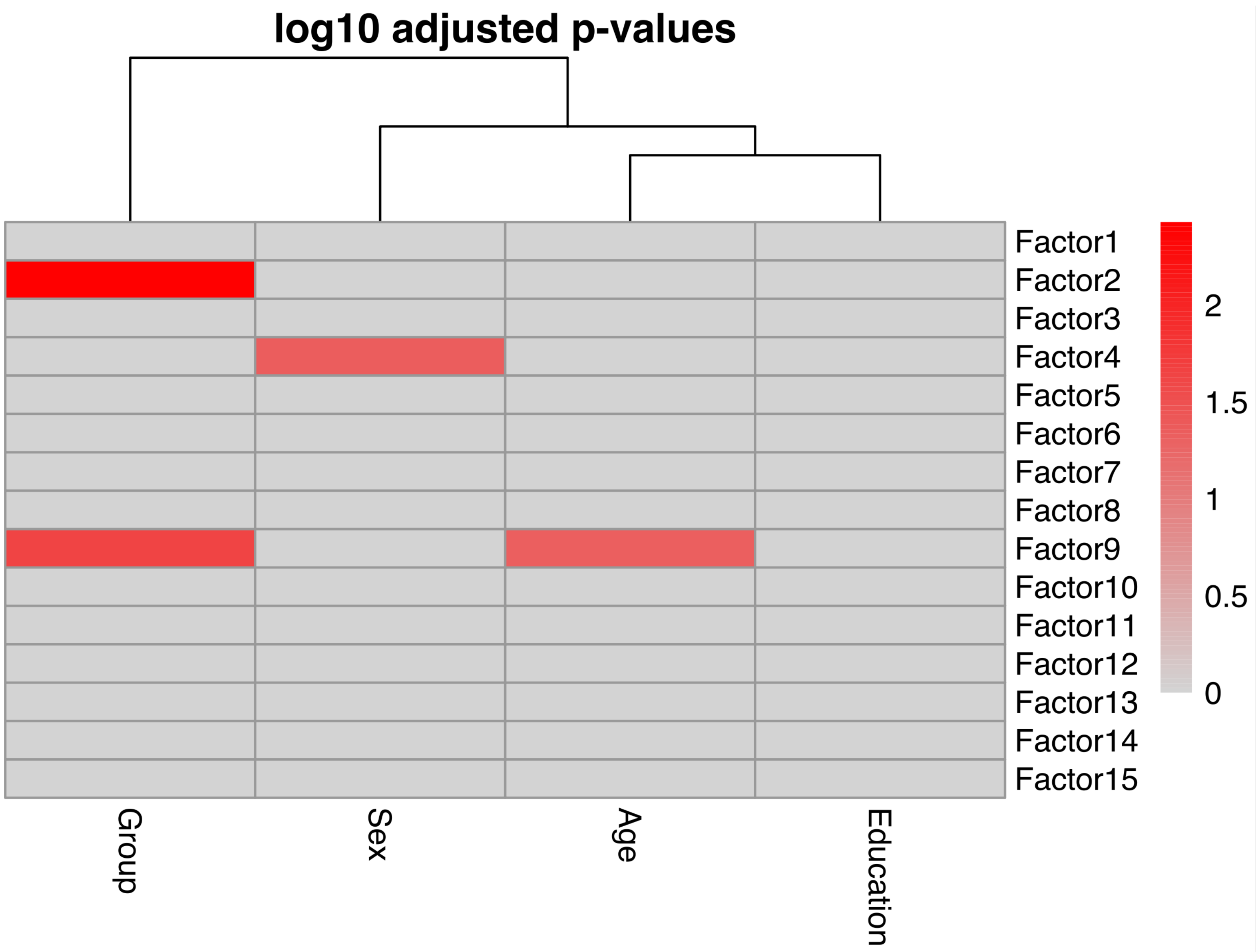


**Supplemental Figure 7.** Association analysis between factor values and covariates. 15 factors were generated in MOFA analysis. Factor 4 was associated with Sex while Factor 9 was associated with both Group and Age. Factor 2 was associated only with Group. There was no significant association between the factors and Education. Corresponding log-transformed *p* values are shown for each significant association. Gray colored boxes indicate no association.

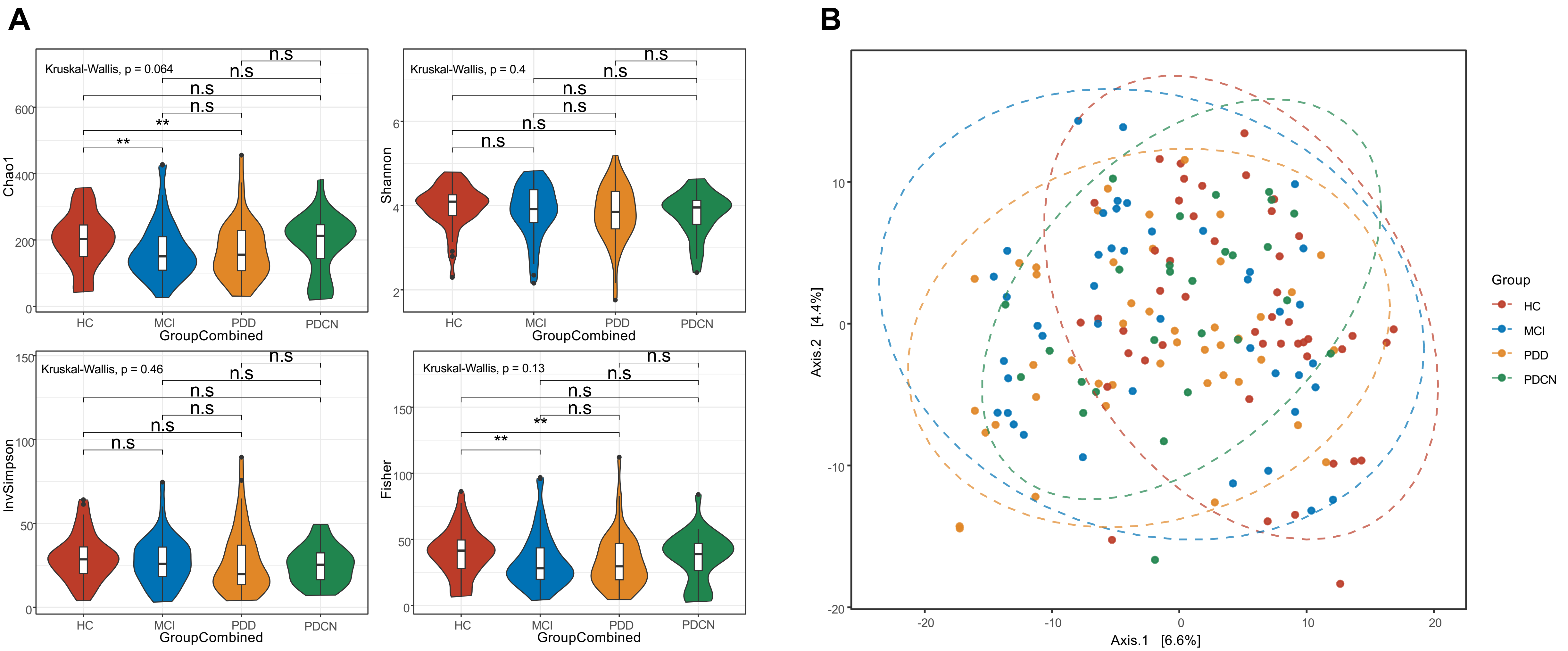


**Supplemental Figure 8.** Alpha and beta diversity comparisons of saliva samples between study groups by 16S rRNA gene amplicon sequencing (including Fleury et al. (2021) samples). (A) Alpha diversity (Chao1, Shannon, InvSimpson, Fisher) comparisons of salivary microbiota samples between study groups. Median estimates compared across study groups using the Kruskal-Wallis test. Boxes represent the interquartile range, lines indicate medians, and whiskers indicate the range. n.s: not significant. (B) Beta diversity comparisons of saliva samples between study groups. PCoA was calculated using Aitchison distance. The ellipses represent a 95% confidence level. Color is indicative of the study group.

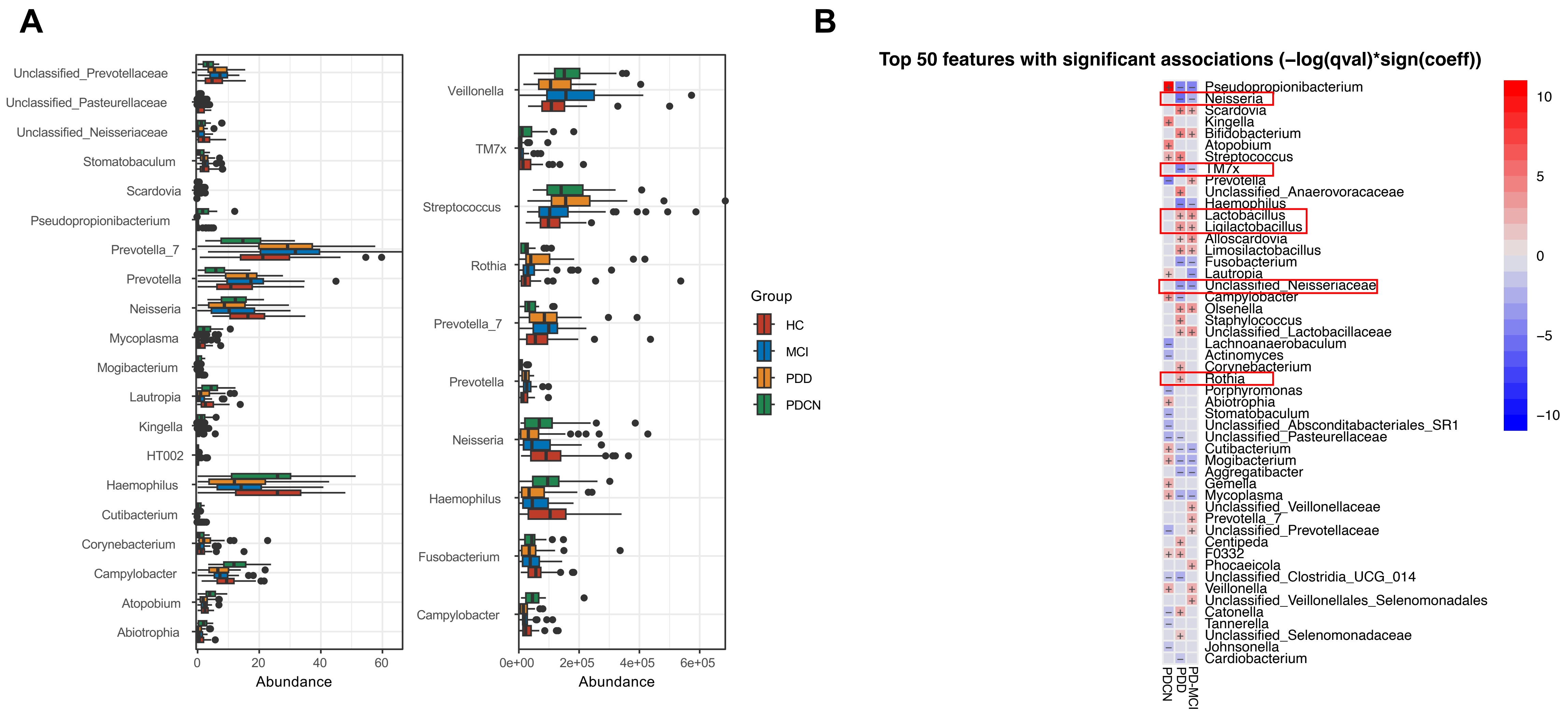


**Supplemental Figure 9. D**ifferential abundance analysis of saliva samples by 16S rRNA gene amplicon sequencing including samples from Fleury et al. (2021) study. (A) Differentially abundant genera detected by ANOVA and LefSe methods ($p<0.05$). Color is indicative of the study group. (B) MaAsLin2 produced heatmap showing the bacterial genera associated with different study groups. The bacterial genera shown in red rectangle are the bacterial genera detected to be associated with PD-MCI and PDD groups before including Fleury et al. (2021) samples.

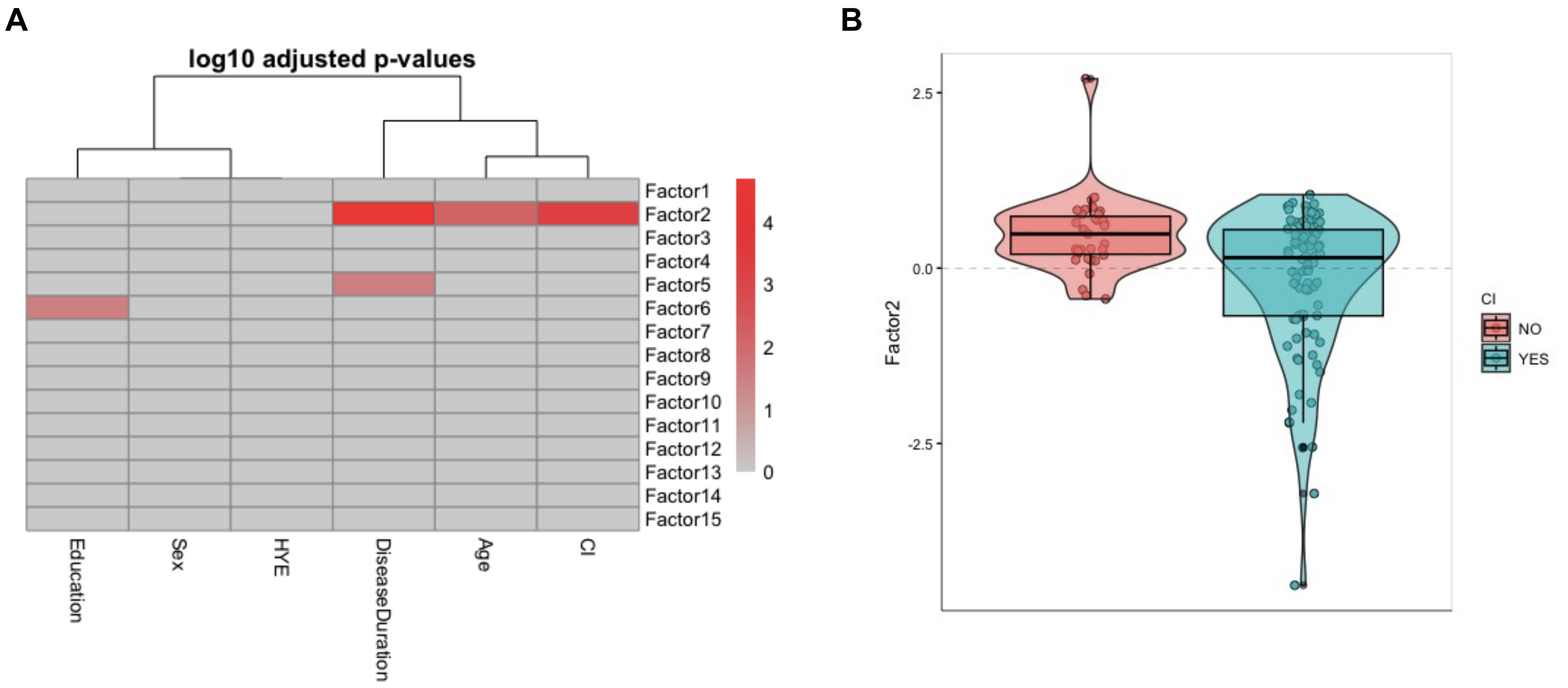


**Supplemental Figure 10.** (A) Association analysis between factor values and covariates. 15 factors were generated in MOFA analysis. Factor 2 was associated with Age, PD Duration and CI. Factor 5 was associated only with PD Duration while Factor 6 was only associated with Education. There was no significant association between the factors and Sex. Corresponding log-transformed *p* values are shown for each significant association. Gray colored boxes indicate no association. (B) Box plot of latent Factor 2 values grouped and colored by CI status.

Supplemental File 1

Results of statistical tests

1. Euclidean distance based comparison for beta diversity (Fleury et al. (2021) and 8 PPD included) (163 samples - AS) (Supplemental Fig. 10B)

Permutation test for adonis under reduced model
Marginal effects of terms
Permutation: free
Number of permutations: 999

adonis2(formula = dist_euclidian ~ GroupCombined, data = metadata, by = "margin", na.action = na.omit)

| | Df | SumOfSqs | R2 | F | Pr(>F) | |
|---|---|---|---|---|---|---|
| GroupCombined | 3 | 7101 | 0.03637 | 2.0006 | 0.001 | *** |
| Residual | 159 | 188116 | 0.96363 | | | |
| Total | 162 | 195217 | 1.00000 | | | |

---
Signif. codes:  0 ‘***’ 0.001 ‘**’ 0.01 ‘*’ 0.05 ‘.’ 0.1 ‘ ’ 1

2. Euclidean distance based comparison for beta diversity (123 samples - AS)

Permutation test for adonis under reduced model
Marginal effects of terms
Permutation: free
Number of permutations: 999

adonis2(formula = dist_euclidean ~ Sex + Age + Education + DiseaseDuration + HYE + CI, data = metadata, by = "margin", na.action = na.omit)

| | Df | SumOfSqs | R2 | F | Pr(>F) | |
|---|---|---|---|---|---|---|
| Sex | 1 | 2789640 | 0.02699 | 2.5156 | 0.006 | ** |
| Age | 1 | 1854706 | 0.01794 | 1.6725 | 0.061 | . |
| Education | 1 | 1279983 | 0.01238 | 1.1542 | 0.260 | |
| DiseaseDuration | 1 | 2973688 | 0.02877 | 2.6815 | 0.008 | ** |
| HYE | 5 | 8188626 | 0.07922 | 1.4768 | 0.012 | * |
| CI | 1 | 2165837 | 0.02095 | 1.9530 | 0.041 | * |
| Residual | 77 | 85389629 | 0.82607 | | | |
| Total | 87 | 103368532 | 1.00000 | | | |

---
Signif. codes:  0 ‘***’ 0.001 ‘**’ 0.01 ‘*’ 0.05 ‘.’ 0.1 ‘ ’ 1

3. Euclidean distance based PERMDISP analysis (123 samples - AS)

Permutation test for homogeneity of multivariate dispersions
Permutation: free
Number of permutations: 999

Response: Distances

```
           Df   Sum Sq Mean Sq      F N.Perm Pr(>F)
Groups      3   345185  115062 0.6286    999  0.606
Residuals 119 21781323  183036
```

4. Bray distance based comparison for beta diversity (123 samples – AS)

```
Permutation test for adonis under reduced model
Marginal effects of terms
Permutation: free
Number of permutations: 999

adonis2(formula = dist_bray ~ Sex + Age + Education +
DiseaseDuration + HYE + CI, data = metadata, by = "margin",
na.action = na.omit)
                Df SumOfSqs      R2      F Pr(>F)
Sex              1   0.4512 0.01784 1.6405  0.014 *
Age              1   0.5580 0.02206 2.0288  0.007 **
Education        1   0.4084 0.01615 1.4848  0.045 *
DiseaseDuration  1   0.3992 0.01578 1.4514  0.056 .
HYE              5   1.6253 0.06426 1.1818  0.066 .
CI               1   0.7075 0.02797 2.5722  0.001 ***
Residual        77  21.1790 0.83737
Total           87  25.2922 1.00000
---
Signif. codes:  0 ‘***’ 0.001 ‘**’ 0.01 ‘*’ 0.05 ‘.’ 0.1 ‘ ’ 1
```

5. Bray distance based PERMDISP analysis (8 PPD included)

```
Permutation test for homogeneity of multivariate dispersions
Permutation: free
Number of permutations: 999

Response: Distances
           Df  Sum Sq   Mean Sq      F N.Perm Pr(>F)
Groups      3 0.01908 0.0063612 0.9835    999  0.409
Residuals 119 0.76968 0.0064679
```

6. Jaccard distance based comparison for beta diversity (123 samples – AS)

```
Permutation test for adonis under reduced model
Marginal effects of terms
Permutation: free
Number of permutations: 999

adonis2(formula = dist_jac ~ Sex + Age + Education + DiseaseDuration
+ HYE + CI, data = metadata, by = "margin", na.action = na.omit)
                Df SumOfSqs      R2      F Pr(>F)
Sex              1   0.4512 0.01784 1.6405  0.017 *
Age              1   0.5580 0.02206 2.0288  0.004 **
Education        1   0.4084 0.01615 1.4848  0.039 *
DiseaseDuration  1   0.3992 0.01578 1.4514  0.052 .
```

```
HYE               5   1.6253 0.06426 1.1818  0.066 .
CI                1   0.7075 0.02797 2.5722  0.001 ***
Residual         77  21.1790 0.83737
Total            87  25.2922 1.00000
---
Signif. codes:  0 ‘***’ 0.001 ‘**’ 0.01 ‘*’ 0.05 ‘.’ 0.1 ‘ ’ 1
```

7. Jaccard distance based PERMDISP analysis (123 samples - AS)

```
Permutation test for homogeneity of multivariate dispersions
Permutation: free
Number of permutations: 999

Response: Distances
           Df  Sum Sq   Mean Sq      F N.Perm Pr(>F)
Groups      3 0.01908 0.0063612 0.9835    999  0.391
Residuals 119 0.76968 0.0064679
```

8. MOFA linear regression factors vs DiseaseDuration (123 samples)

```
Call:
lm(formula = value ~ Sex + Age + Education + DiseaseDuration,
    data = factors)

Residuals:
    Min      1Q  Median      3Q     Max
-3.9183 -0.3070  0.2152  0.5473  2.3174

Coefficients:
                 Estimate Std. Error t value Pr(>|t|)
(Intercept)      1.690322   0.702161   2.407 0.017690 *
SexM             0.291399   0.175179   1.663 0.098996 .
Age             -0.019107   0.010432  -1.831 0.069665 .
Education       -0.023989   0.018091  -1.326 0.187511
DiseaseDuration -0.006074   0.001591  -3.819 0.000219 ***
---
Signif. codes:  0 ‘***’ 0.001 ‘**’ 0.01 ‘*’ 0.05 ‘.’ 0.1 ‘ ’ 1

Residual standard error: 0.9008 on 113 degrees of freedom
  (5 observations deleted due to missingness)
Multiple R-squared:  0.1883,    Adjusted R-squared:  0.1596
F-statistic: 6.555 on 4 and 113 DF,  p-value: 8.825e-05
```

9. MOFA linear regression factors vs Group (123 samples)

```
Call:
lm(formula = value ~ Sex + Age + Education + Group, data = factors)

Residuals:
    Min      1Q  Median      3Q     Max
-4.1648 -0.3774  0.1799  0.6099  2.3302
```

```
Coefficients:
            Estimate Std. Error t value Pr(>|t|)  
(Intercept)  1.67255    0.73085   2.288   0.0239 *
SexM         0.36699    0.18412   1.993   0.0486 *
Age         -0.01893    0.01129  -1.677   0.0963 .
Education   -0.01912    0.01933  -0.989   0.3246  
GroupMCI    -0.64067    0.25298  -2.532   0.0127 *
GroupPDD    -0.64672    0.28487  -2.270   0.0251 *
GroupPPD    -0.32521    0.41947  -0.775   0.4398  
---
Signif. codes:  0 ‘***’ 0.001 ‘**’ 0.01 ‘*’ 0.05 ‘.’ 0.1 ‘ ’ 1

Residual standard error: 0.9318 on 115 degrees of freedom
  (1 observation deleted due to missingness)
Multiple R-squared:  0.137,     Adjusted R-squared:  0.09199 
F-statistic: 3.043 on 6 and 115 DF,  p-value: 0.008454
```

10. Euclidean distance based comparison for beta diversity (115 samples - AS)

```
Permutation test for adonis under reduced model
Marginal effects of terms
Permutation: free
Number of permutations: 999

adonis2(formula = dist_euclidean ~ Age + Sex + Education + Group, data = metadata, by = "margin")
           Df  SumOfSqs      R2      F Pr(>F)   
Age         1    986064 0.00883 1.0280  0.388   
Sex         1   1970254 0.01765 2.0540  0.007 **
Education   1    765541 0.00686 0.7981  0.713   
Group       2   2836862 0.02541 1.4787  0.021 * 
Residual  109 104554195 0.93642                 
Total     114 111653154 1.00000                 
---
Signif. codes:  0 ‘***’ 0.001 ‘**’ 0.01 ‘*’ 0.05 ‘.’ 0.1 ‘ ’ 1
```

11. Euclidean distance based PERMDISP analysis (115 samples - AS)

```
Permutation test for homogeneity of multivariate dispersions
Permutation: free
Number of permutations: 999

Response: Distances
           Df   Sum Sq Mean Sq      F N.Perm Pr(>F)
Groups      2   224198  112099 0.6373    999  0.528
Residuals 112 19699956  175892
```

12. Bray distance based comparison for beta diversity (115 samples - AS)

```
Permutation test for adonis under reduced model
Marginal effects of terms
Permutation: free
```

```
Number of permutations: 999

adonis2(formula = dist_bray ~ Age + Sex + Education + Group, data =
metadata, by = "margin")
           Df SumOfSqs      R2      F Pr(>F)
Age         1    0.428 0.01296 1.5094  0.034 *
Sex         1    0.486 0.01474 1.7167  0.015 *
Education   1    0.345 0.01046 1.2179  0.188
Group       2    0.779 0.02361 1.3752  0.024 *
Residual  109   30.885 0.93585
Total     114   33.002 1.00000
---
Signif. codes:  0 ‘***’ 0.001 ‘**’ 0.01 ‘*’ 0.05 ‘.’ 0.1 ‘ ’ 1
```

13. Bray distance based PERMDISP analysis (115 samples - AS)

```
Permutation test for homogeneity of multivariate dispersions
Permutation: free
Number of permutations: 999

Response: Distances
           Df  Sum Sq   Mean Sq      F N.Perm Pr(>F)
Groups      2 0.00913 0.0045659 0.8515    999  0.429
Residuals 112 0.60060 0.0053625
```

14. Jaccard distance based comparison for beta diversity (115 samples - AS)

```
Permutation test for adonis under reduced model
Marginal effects of terms
Permutation: free
Number of permutations: 999

adonis2(formula = dist_jac ~ Age + Sex + Education + Group, data =
metadata, by = "margin")
           Df SumOfSqs      R2      F Pr(>F)
Age         1    0.428 0.01296 1.5094  0.038 *
Sex         1    0.486 0.01474 1.7167  0.013 *
Education   1    0.345 0.01046 1.2179  0.167
Group       2    0.779 0.02361 1.3752  0.026 *
Residual  109   30.885 0.93585
Total     114   33.002 1.00000
---
Signif. codes:  0 ‘***’ 0.001 ‘**’ 0.01 ‘*’ 0.05 ‘.’ 0.1 ‘ ’ 1
```

15. Jaccard distance based PERMDISP analysis (115 samples - AS)

```
Permutation test for homogeneity of multivariate dispersions
Permutation: free
Number of permutations: 999

Response: Distances
           Df  Sum Sq   Mean Sq      F N.Perm Pr(>F)
```

```
Groups      2 0.00913 0.0045659 0.8515    999  0.447
Residuals 112 0.60060 0.0053625
```

16. Euclidean distance based comparison for beta diversity (115 samples - MP)

```
Permutation test for adonis under reduced model
Marginal effects of terms
Permutation: free
Number of permutations: 999

adonis2(formula = euc_dist_adj ~ ProtBatch + Group, data = metadata,
by = "margin")
           Df SumOfSqs      R2      F Pr(>F)
ProtBatch   4       75 0.00219 0.0604  1.000
Group       2      701 0.02553 1.1331  0.025 *
Residual  108    33424 0.97849
Total     114    34158 1.00000
---
Signif. codes:  0 '***' 0.001 '**' 0.01 '*' 0.05 '.' 0.1 ' ' 1
```

17. Euclidean distance based PERMDISP analysis (115 samples - MP)

```
Permutation test for homogeneity of multivariate dispersions
Permutation: free
Number of permutations: 999

Response: Distances
           Df  Sum Sq Mean Sq      F N.Perm Pr(>F)
Groups      2  269.61 134.804 7.3619    999  0.002 **
Residuals 112 2050.85  18.311
---
Signif. codes:  0 '***' 0.001 '**' 0.01 '*' 0.05 '.' 0.1 ' ' 1
```

18. Euclidean distance based comparison for beta diversity (115 samples - MP - adj)

```
Permutation test for adonis under reduced model
Marginal effects of terms
Permutation: free
Number of permutations: 999

adonis2(formula = euc_dist_adj ~ ProtBatch + Age + Sex + Education +
Group, data = metadata, by = "margin")
           Df SumOfSqs      R2      F Pr(>F)
ProtBatch   4       94 0.00274 0.0757  1.000
Age         1      284 0.00831 0.9168  0.685
Sex         1      357 0.01046 1.1537  0.139
Education   1      223 0.00653 0.7205  0.985
Group       2      661 0.01935 1.0673  0.082
Residual  105    32518 0.95199
Total     114    34158 1.00000
```

19. Euclidean distance based comparison for beta diversity (123 samples - MP - adj)

```
Permutation test for adonis under reduced model
Marginal effects of terms
Permutation: free
Number of permutations: 999

adonis2(formula = euc_dist_adj ~ Sex + Age + Education +
DiseaseDuration + HYE + CI, data = metadata, by = "margin",
na.action = na.omit)
                Df SumOfSqs      R2      F Pr(>F)
Sex              1    294.7 0.01133 0.9814  0.501
Age              1    218.7 0.00840 0.7281  0.968
Education        1    343.8 0.01322 1.1450  0.188
DiseaseDuration  1    400.1 0.01538 1.3323  0.067 .
HYE              1    251.4 0.00966 0.8370  0.828
CI               1    146.4 0.00563 0.4876  0.999
Residual        81  24323.6 0.93495
Total           87  26015.8 1.00000
---
Signif. codes:  0 ‘***’ 0.001 ‘**’ 0.01 ‘*’ 0.05 ‘.’ 0.1 ‘ ’ 1
```

20. Euclidean distance based PERMDISP analysis (123 samples – MP)

```
Permutation test for homogeneity of multivariate dispersions
Permutation: free
Number of permutations: 999

Response: Distances
           Df Sum Sq Mean Sq     F N.Perm Pr(>F)
Groups      1   74.2  74.219 2.819    999  0.085 .
Residuals 121 3185.7  26.328
---
Signif. codes:  0 ‘***’ 0.001 ‘**’ 0.01 ‘*’ 0.05 ‘.’ 0.1 ‘ ’ 1
```
