# Supplementary material for: Metaproteogenomic analysis of saliva samples from Parkinson’s disease patients with cognitive impairment
Source: NPJ Biofilms Microbiomes. 2023 Nov 18;9:86. doi: 10.1038/s41522-023-00452-x (PMC10657361; doi:10.1038/s41522-023-00452-x)
Supplement: Supplementary file 2 — Reporting Summary [file 41522_2023_452_MOESM2_ESM.pdf]

nature portfolio

Corresponding author(s): Suleyman Yildirim

Last updated by author(s): Jul 18, 2023

Reporting Summary

Nature Portfolio wishes to improve the reproducibility of the work that we publish. This form provides structure for consistency and transparency in reporting. For further information on Nature Portfolio policies, see our Editorial Policies and the Editorial Policy Checklist.

Statistics

For all statistical analyses, confirm that the following items are present in the figure legend, table legend, main text, or Methods section.

n/a	Confirmed	
☐	☒	The exact sample size (*n*) for each experimental group/condition, given as a discrete number and unit of measurement
☐	☒	A statement on whether measurements were taken from distinct samples or whether the same sample was measured repeatedly
☐	☒	The statistical test(s) used AND whether they are one- or two-sided<br>*Only common tests should be described solely by name; describe more complex techniques in the Methods section.*
☐	☒	A description of all covariates tested
☐	☒	A description of any assumptions or corrections, such as tests of normality and adjustment for multiple comparisons
☐	☒	A full description of the statistical parameters including central tendency (e.g. means) or other basic estimates (e.g. regression coefficient) AND variation (e.g. standard deviation) or associated estimates of uncertainty (e.g. confidence intervals)
☐	☒	For null hypothesis testing, the test statistic (e.g. *F*, *t*, *r*) with confidence intervals, effect sizes, degrees of freedom and *P* value noted<br>*Give P values as exact values whenever suitable.*
☒	☐	For Bayesian analysis, information on the choice of priors and Markov chain Monte Carlo settings
☒	☐	For hierarchical and complex designs, identification of the appropriate level for tests and full reporting of outcomes
☒	☐	Estimates of effect sizes (e.g. Cohen's *d*, Pearson's *r*), indicating how they were calculated

Our web collection on statistics for biologists contains articles on many of the points above.

Software and code

Policy information about availability of computer code

Data collection	Excel (Microsoft Office), SPSS (IBM)
Data analysis	Excel (Microsoft Office), R packages, Progenesis-QI (Waters), ProPHAnE (PMID:32859984, Nephele (PMID:29028892). We did not use in-house coded computer language scripts.

For manuscripts utilizing custom algorithms or software that are central to the research but not yet described in published literature, software must be made available to editors and reviewers. We strongly encourage code deposition in a community repository (e.g. GitHub). See the Nature Portfolio guidelines for submitting code & software for further information.

Data

Policy information about availability of data

All manuscripts must include a data availability statement. This statement should provide the following information, where applicable:

- Accession codes, unique identifiers, or web links for publicly available datasets
- A description of any restrictions on data availability
- For clinical datasets or third party data, please ensure that the statement adheres to our policy

The raw 16S rRNA gene amplicon sequencing data produced in this study have been deposited in the NCBI Sequence Read Archive database under accession no.

PRJNA913682. Metaproteomics data generated in this study are not publicly available to preserve individuals' privacy under the European General Data Protection Regulation

Research involving human participants, their data, or biological material

Policy information about studies with human participants or human data. See also policy information about sex, gender (identity/presentation), and sexual orientation and race, ethnicity and racism.

Reporting on sex and gender	Sex and/or gender was determined based on self reporting and all reported biological sex, none preferred gender-based option.
Reporting on race, ethnicity, or other socially relevant groupings	Race, ethnicity, or other socially relevant groupings were not mentioned, implicitly or explicitly, in our manuscript. We added a list of adjusted confounding factors as supplemental file.
Population characteristics	The following covariates were adjusted in our analyses: Sex, Age, Education, Disease Duration, HYE, and Cognition status
Recruitment	Clinicians in this study invited the patients they routinely follow (or new diagnosis) to participate in the study, if the patient meets the exclusion/inclusion criteria.
Ethics oversight	The study was approved by the ethics committee of the Istanbul Medipol University with authorization number 10840098-604.01.01-E.3958, and informed consent was obtained from all participants.

Note that full information on the approval of the study protocol must also be provided in the manuscript.

Field-specific reporting

Please select the one below that is the best fit for your research. If you are not sure, read the appropriate sections before making your selection.

☒ Life sciences ☐ Behavioural & social sciences ☐ Ecological, evolutionary & environmental sciences

For a reference copy of the document with all sections, see nature.com/documents/nr-reporting-summary-flat.pdf

Life sciences study design

All studies must disclose on these points even when the disclosure is negative.

Sample size	We performed power analysis using micropower R package (https://github.com/brendankelly/micropower) using the preliminary data we collected, which indicated that n=30 participants per group and N=90 for 3 groups would be sufficient (80% power, based on the assumptions of 20% drop-out, 20% death rate). The size of the cohort in this study was N=115.
Data exclusions	No data were excluded from the analyses.
Replication	This is a cross sectional study and each group includes several participants, N=115 samples in total. We separately run qPCR to confirm abundances of most abundant taxa (data not shown).
Randomization	Samples from each group were randomly picked from the freezers for wet lab processing to avoid incorporating systematic bias
Blinding	Blinding was not possible because the participants were recruited based on their clinical diagnosis.

Reporting for specific materials, systems and methods

We require information from authors about some types of materials, experimental systems and methods used in many studies. Here, indicate whether each material, system or method listed is relevant to your study. If you are not sure if a list item applies to your research, read the appropriate section before selecting a response.

Materials & experimental systems

n/a	Involved in the study	
☒	☐	Antibodies
☒	☐	Eukaryotic cell lines
☒	☐	Palaeontology and archaeology
☒	☐	Animals and other organisms
☐	☒	Clinical data
☒	☐	Dual use research of concern
☒	☐	Plants

Methods

n/a	Involved in the study	
☒	☐	ChIP-seq
☒	☐	Flow cytometry
☒	☐	MRI-based neuroimaging

Clinical data

Policy information about clinical studies

All manuscripts should comply with the ICMJE guidelines for publication of clinical research and a completed CONSORT checklist must be included with all submissions.

Clinical trial registration	This was not a clinical trial but observational study (no intervention)
Study protocol	N/A
Data collection	Tertiary hospitals for sample collection and research lab for molecular and bioinformatic analysis.
Outcomes	Cross sectional study
